# Characterisation, symptom pattern and symptom clusters from a retrospective cohort of Long COVID patients in primary care in Catalonia

**DOI:** 10.1186/s12879-023-08954-x

**Published:** 2024-01-15

**Authors:** Gemma Torrell, Diana Puente, Constanza Jacques-Aviñó, Lucia A. Carrasco-Ribelles, Concepció Violán, Tomás López-Jiménez, Veronica Royano, Alba Molina Cantón, Laura Medina-Perucha, Israel Rodríguez-Giralt, Anna Berenguera

**Affiliations:** 1 Centre d’Atenció Primària Les Indianes, Gerència Territorial de Barcelona, Institut Català de la Salut, Barcelona, Spain; 2 Fundació Institut Universitari per a la recerca a l’Atenció Primària de Salut Jordi Gol i Gurina (IDIAPJGol), Barcelona, Spain; 3 Universitat Autònoma de Barcelona, Cerdanyola del Vallès, Spain; 4 Network for Research on Chronicity, Primary Care, and Health Promotion (RICAPPS) (RD21/0016/0029) Insitituto de Salud Carlos III, Madrid, Spain; 5 Unitat de Suport a la Recerca Metropolitana Nord, Institut Universitari d’Investigació en Atenció Primària Jordi Gol (IDIAP Jordi Gol), Mataró, Spain; 6 Grup de REcerca en Impacte de les Malalties Cròniques i les seves Trajectòries (GRIMTra), (2021 SGR 01537), Institut Universitari d’Investigació en Atenció Primària Jordi Gol (IDIAPJGol), 08008 Barcelona, Spain; 7 Direcció d’Atenció Primària Metropolitana Nord Institut Català de Salut, Barcelona, Spain; 8 Universitat Politècnica de Catalunya BarcelonaTech (UPC), Barcelona, Spain; 9 Members of the Col·lectiu d’Afectades i Afectats Persistents per COVID-19 a Catalunya, Barcelona, Spain; 10 Internet Interdisciplinary Institute (IN3), Universitat Oberta de Catalunya, Barcelona, Spain

**Keywords:** SARS-CoV-2, Long COVID, Post-COVID-19 syndrome, Cluster analysis, Primary health care, Participatory research

## Abstract

**Background:**

Around 10% of people infected by SARS-COV-2 report symptoms that persist longer than 3 months. Little has been reported about sex differences in symptoms and clustering over time of non-hospitalised patients in primary care settings.

**Methods:**

This is a descriptive study of a cohort of mainly non-hospitalized patients with a persistence of symptoms longer than 3 months from the clinical onset in co-creation with the Long Covid Catalan affected group using an online survey. Recruitment was from March 2020 to June 2021. Exclusion criteria were being admitted to an ICU, < 18 years of age and not living in Catalonia. We focused on 117 symptoms gathered in 18 groups and performed cluster analysis over the first 21 days of infection, at 22–60 days, and ≥ 3 months.

**Results:**

We analysed responses of 905 participants (80.3% women). Median time between symptom onset and the questionnaire response date was 8.7 months. General symptoms (as fatigue) were the most prevalent with no differences by sex, age, or wave although its frequency decreased over time (from 91.8 to 78.3%). Dermatological (52.1% in women, 28.5% in men), olfactory (34.9% women, 20.9% men) and neurocognitive symptoms (70.1% women, 55.8% men) showed the greatest differences by sex. Cluster analysis showed five clusters with a predominance of *Taste & smell* (24.9%) and *Multisystemic* clusters (26.5%) at baseline and *_Multisystemic (34.59%)* and *Heterogeneous (24.0%)* at ≥3 months. The *Multisystemic* cluster was more prevalent in men. The *Menstrual* cluster was the most stable over time, while most transitions occurred from the *Heterogeneous* cluster to the *Multisystemic* cluster and from *Taste & smell* to *Heterogeneous*.

**Conclusions:**

General symptoms were the most prevalent in both sexes at three-time cut-off points. Major sex differences were observed in dermatological, olfactory and neurocognitive symptoms. The increase of the *Heterogeneous* cluster might suggest an adaptation to symptoms or a non-specific evolution of the condition which can hinder its detection at medical appointments. A carefully symptom collection and patients’ participation in research may generate useful knowledge about Long Covid presentation in primary care settings.

**Supplementary Information:**

The online version contains supplementary material available at 10.1186/s12879-023-08954-x.

## Background

From March 2020 onwards, many people infected with SARS-COV-2 who were never hospitalised during the acute phase of the disease presented with persisting symptoms three or more months after symptom onset. At the beginning of pandemic, little attention was paid to mild or moderate symptoms. There was only a single story about what COVID-19 was: a potentially deadly respiratory disease [1]. People with mild or moderate COVID-19 who developed persistent symptoms were invisible in the eyes of the health system and their immediate surroundings. They gathered through social media in a number of countries to raise awareness about their condition in the scientific community (who were sceptical about its existence) and began to produce knowledge about it [2] before the first scientific study was published [3]. Thus, the first studies were created based on self-reported data. This condition, referred to as Long COVID by patients [4] and renamed as Post-COVID Condition by the World Health Organisation (WHO) [5], has been estimated to affect 10–50% of people infected with SARS-COV-2 depending on the initial clinical spectrum of infection [6–8].

Long COVID has been described as a multisystemic condition [9] with many fluctuating symptoms at different levels of intensity over time which causes different levels of episodic (or long-term) impairment on a person’s ability to do normal day-to-day activities [9, 10]. Long COVID constitutes a long-term condition or evolution of COVID-19 independent of the severity of the acute disease [11]. However, the mechanisms related to the persistence of symptoms are unknown being the main hypothesis investigated: persistence of virus, chronic inflammation with blood clotting, existence of autoantibodies, microbiota dysbiosis, tissue damage and dysfunctional neurological signalling [12–18]. Other studies have found that low cortisol levels may be a biomarker for Long COVID [19]. Although there are many ongoing studies trying to find a specific biomarker for Long COVID, as yet there is no consistent evidence available.

Long COVID has been described to be more prevalent in women than in men and at about middle age [2, 9, 20, 21]. Specifically, some articles point out that Body Mass Index (BMI), female sex, increasing age and having comorbidities [22] are risk factors for Long COVID. Other studies report that the presence of five symptoms such as fatigue, headache, dyspnoea, hoarse voice and myalgia at the first week of the disease can also be risk factors for Long COVID [20, 22, 23]. As some studies point out, however, gender differences may not only be related to differences in the prevalence and symptomatology of the condition but also to broader social and cultural factors that affect how individuals are perceived and treated by others [24].

Some studies have described symptoms, categorised them in domains, grouped them in clusters and then observed their evolution over time, suggesting the existence of different phenotypes which can help to identify the mechanisms involved and also different care needs [21, 25–27].

Long COVID symptoms can be identified through the reporting of symptoms recorded by health professionals in the EHR or by symptoms self-reported by people affected by Long COVID through public participation, as this study does [28].

Some studies have identified different trajectories of the evolution of post-COVID-19 conditions. For example, one study identified three trajectories: “high persistent symptoms,” “rapidly decreasing symptoms,” and “slowly decreasing symptoms” [29]. Another study found that COVID-19 symptoms persisted for 1 year after illness onset, even in some individuals with mild disease, and that female sex and obesity were associated with symptoms persistence [30].

There are studies that have identified the evolution of symptoms and trajectories over time. However, little is known about the study of symptom evolution since the onset of the disease. Access to this information is only possible in studies conducted since the beginning of the SARS-CoV-2 pandemic.

Thus, much has been described about the symptoms of Long COVID but there is still much to learn about the evolution of persistent COVID-19 symptoms, also known as post-COVID conditions (PCCs) or Long COVID.

This study aims to add knowledge about Long COVID symptoms and their evolution over time and to highlight the co-participatory research work between patients and primary care professionals.

## Methods

### Design

It consists in a retrospective cohort of adults.

### Study population

This study was co-created with people belonging to the Long COVID group in Catalonia [31] that involved participants with Long COVID symptoms in Catalonia (Spain).

Inclusion criteria were being ≥18 years old, living in Catalonia and having symptoms that lasted more than 3 months after suspected or confirmed (by a positive Polymerase Chain Reaction or Rapid Antigen Test) SARS-COV-2 infection and agreeing to participate and confirming their availability to answer surveys. People who had been hospitalised in an ICU (Intensive Care Unit) were excluded. The 3 month inclusion criterion was based on the available information provided by Greenhalgh et al. in August 2020 [32].

Recruiting was performed through people belonging to the Catalan Long COVID group through social media (Twitter, blog, WhatsApp group) and by snowball sampling. It was publicised through a webinar for primary care professionals (doctors, nurses, social workers) working for health providers in Catalonia to recruit more participants.

Recruiting was opened on 3rd December 2020 and closed on 30th June 2021. However, cases that were diagnosed during the first and second wave were also collected.

People were asked to report their symptoms at the first 21 days from symptom onset (baseline), at 22–60 days and at ≥3 months from the initial diagnosis. These cut-off points were based on available studies in 2020, about the average time for recovery from mild COVID-19 and the cut-off point used by patient led reports [33].

### Data source

This paper looks at the recruitment questionnaire of this study and the variables related to sociodemographic data, clinical data and symptoms out of 40 variables included in the questionnaire that supply information about various domains (not included in this analysis) such as quality of life, use of the health system and others.

The variables were collected by a self-reported questionnaire initially performed by people affected based on their own questions about their condition and finally worked out together with a primary care doctor and a research group from the Institut Universitari d’Investigació en Atenció Primària (IDIAPJ Gol).

A group belonging to the *Col·lectiu d’Afectades i Afectats persistents per COVID-19 a Catalunya* [31] participated in the design of the study and two of them in the discussions of the results, sharing their experiences and points of view and enriching each part of the project.

Data were hosted on the REDCap (Research Electronic Data Capture) platform, allowing participants to enter their data while retaining anonymity and protection. REDCap is a secure, web-based software platform designed to collect data for research studies providing: 1) an intuitive interface for validated data capture; 2) audit trails for tracking data manipulation and export procedures; 3) automated export procedures for seamless data downloads to common statistical packages, and 4) procedures for data integration and interoperability with external sources [34, 35].

### Variables

The main variable was symptoms. In total, 117 symptoms were collected; their attributes were YES/NO.

Symptoms were gathered by systems and creating a new variable for each system: dermatological, ophthalmological, urological, sexually related, menstruation related, general (including fatigue and fever), rheumatologic, neurological (including headache and insomnia), digestive, gyneacological, neurocognitive, cardiac, respiratory, upper airway, ear, nose, and throat (ENT), disautonomic, olfactory and altered taste and smell based on clinical intuition. All of them were stratified by sex (women, men), age (18–34, 35–49, 50–64, ≥65 years) and wave. Information about what symptoms each system contains can be found in the Supplementary Data 1 (SD1).

Co-variables were date of self-reporting of the initial questionnaire, and sociodemographic data such as sex, date of birth, weight, and height. Clinical data related to date of symptom onset, type of symptoms, previous comorbidities, previous treatments and diagnostic tests were also included.

The dates of the pandemic waves were gathered from Ministerio de Sanidad data published in the reports by the Red Nacional de Vigilancia Epidemiológica (RENAVE) establishing the following periods: first wave from 13th March 2020 to 21st June 2020, second wave from 22nd June 2020 to 6th December 2020 and third wave from 7th December 2020 to 14th March 2021 [36].

Symptom perception evolution was self-reporting, and its variable was created through six graphics and definitions constructed by patients themselves and following the trends of the symptoms they had been experiencing and noting down in a diary since the beginning of these symptoms (Fig. S1).

### Data analysis

An initial descriptive analysis of the included population was performed using mean (standard deviation) and median (interquartile range) for quantitative variables and percentages for categorical variables. To assess differences between sex and age, the t-test or the U Mann-Whitney test for quantitative variables and the Chi-squared test for qualitative variables were performed. A Trend test was performed to assess differences between symptoms by system at the three cut-offs (Table S1 and Fig. S2). Stratified analysis for symptom length at < 21 days (baseline), 22–60 days and ≥ 3 months days was performed.

To identify clusters of symptoms by Long COVID system, PCAmix [37] transformation of the data was performed prior to applying fuzzy c-means to reduce dimensionality. In this reduction, symptoms by systems, age and sex of the individuals were considered, leaving a total of four dimensions after applying the Karlis-Saporta-Spinaki criterion [37, 38]. Fuzzy c-means is a soft clustering technique that relates the symptoms by system of each individual at each time point (i.e., < 21 days, 20–60 days, and ≥ 3 months) to a different cluster through membership probability [39]. Having each participant’s time point assigned to a cluster made it possible to draw each individual’s course in terms of patterns of system affection due to Long COVID over time. The number of clusters (from 2 to 8) and degree of fuzziness (from 1.1 to 1.8, per 0.1) were chosen through validation indices calculated 100 times in order to account for the random nature of the clustering initialisation. Once the clusters had been identified, symptoms by system at each time point were assigned to the cluster for which they had the highest membership probability. The clusters were described through the calculation of observed/expected ratios (OE ratios), which compares the prevalence of the symptom by system in each cluster with that in the study population. In addition, exclusivity was calculated as the percentage of records presented by each system divided by the total number of records with that system in the study population. A system with an OE > 2.5% or an exclusivity > 30% was considered as characteristic of the cluster and used to name the cluster. This approach has already been used in other studies [38, 40–43]. R v 4.0.2. was used to conduct the clustering analysis.

## Results

**Fig. 1:**
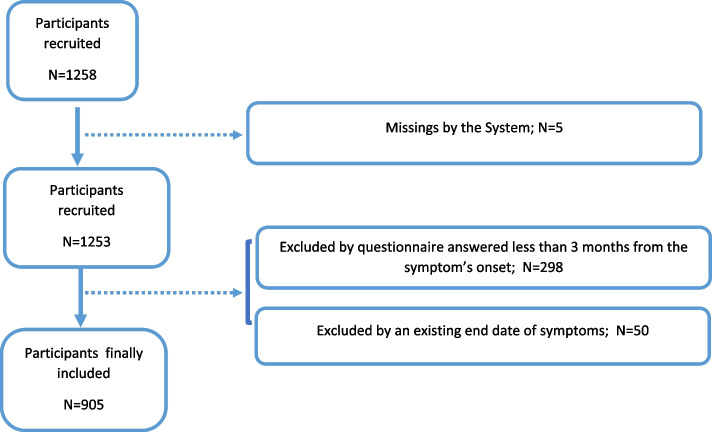
Flow chart of the study population

From 1258 respondents, we excluded those who had less than 3 months from the beginning of symptom onset to the enrolment date (*n* = 298), those who were missing a symptoms variable (*n* = 5) and those who reported an end date of symptoms of less than 3 months from the symptom’s onset (*N* = 47) (Fig. 1).

Finally, 905 respondents who had symptoms for 3 or more months from symptom onset (80.3% women, 19.0% men and 0.7% non-binary) were included. Median age was 46.0 years, 57.1% had comorbidities and 51.8% reported not taking any chronic treatment. Median Body Mass Index (BMI) was 24.2%, a third of respondents were non-smokers (32.7%) and 37.1% did physical exercise 2–3 times a week before SARS-COV-2 infection. 3.3% (30 from the total of 905) of participants (4.6% of men and 3.02% of women) reported an end date of their symptoms, which showed a median of 184 days (p25-p75 of 156.2 days to 389.2 days) since the onset of symptoms higher in men (184 days) than in women (183 days). Characteristics of the self-reported cohort are presented in Table 1 and characteristics of the “end date of symptoms cohort” are in Table S2.

**Table 1:** Socio-demographical and clinical characteristics of the self-reported cohort

Characteristics	Total N (%)	Female N(%)	Male N(%)	*p*-value
**Gender**	905 (100)	727 (80.3)	172 (19.0)	
**Age (years)**				
Median (P25-P75)	46.0 (40–54)	46.0 (39–53)	49.0 (42–56)	
**Source of income**				
Contract worker	696 (76.9)	571 (78.5)	120 (69.8)	0.046
Independent worker with contribution	70 (7.7)	46 (6.3)	24 (14.0)	0.003
Informal worker (no contract nor contribution)	19 (2.1)	16 (2.2)	3 (1.7)	0.873
Unemployment benefit or other benefits	25 (2.8)	18 (2.5)	7 (4.1)	0.475
Unemployed without benefit nor social benefit	9 (1.0)	7 (1.0)	2 (1.2)	0.943
Household chores or caregiver	13 (1.4)	12 (1.7)	1 (0.6)	0.546
Student	45 (5,0)	34 (4,7)	11 (6.4)	0.553
Other	74 (8.2)	58 (8.0)	15 (8.7)	0.711
**Health worker**^**a**^				0.000
No	611 (67.8)	462 (63.9)	145 (84.3)	
Yes	290 (36.1)	261 (36.1)	27 (15.7)	
**Previous physical activity**^**a**^				0.326
Everyday	152 (16.9)	123 (17.0)	28 (16.5)	
2–3 times a week	334 (37.1)	268 (37.0)	65 (38.2)	
< 2–3 times a week	202 (22.4)	163 (22.5)	39 (22.9)	
No practice	213 (23.6)	171 (23.6)	38 (22.4)	
**Smoking hàbit**^**b**^				
Smoker	78 (8,6)	68 (9.4)	9 (5.3)	0.180
Non-smoker	296 (32.7)	241 (33.1)	54 (31.6)	0.649
Ex-smoker	228 (25.2)	157 (21.6)	69 (40.4)	**0.000**
**BMI (kg/m2)**				
Median (P25-P75)	24.2 (21.5–27.8)	23.8 (21.2–27.3)	25.3 (23.1–28.7)	
**Comorbidities**				
No	388 (42.9)	287 (40.9)	88 (51.2)	0.046
Yes	517 (57.1)	430 (59.1)	84 (48.8)	
**Previous treatments**				
Yes	436 (48.2)	363 (49.9)	70 (40.7)	0.093
No	469 (51.8)	364 (50.1)	102 (59.3)	
**Hospitalization**^**a**^				
Yes	142 (15.7)	102 (14.0)	39 (22.7)	
**Positive at any time by PCR or TAR**^**a**^				0.533
Never positive	437 (48.5)	345 (47.6)	89 (52.4)	
Sometime positive	464 (51.5)	380 (52.4)	81 (47.6)	
**WAVE**^**c**^				0.981
First wave	442 (60.0)	361 (60.1)	78 (60.0)	
Second wave	253 (34.3)	205 (34.1)	45 (34.6)	
Third wave	41 (5.6)	34 (5.7)	7 (5.4)	
Fourth wave	1 (0.1)	1 (0.2)	0 (0)	
**Vaccinated (probable)**^**d**^				0.903
< 27/12/2020	882 (97.5)	708 (97.4)	168 (97.7)	
> 27/12/2020	23 (2,5)	19 (2.6)	4 (2.3)	
**Median number of symptoms by period (p25-p75)**				
< 21 days	24 (15–37)	25 (16–38)	20 (11–33)	
22–60 days	20 (9–32)	21 (10–34)	16 (7–28)	
≥3 months	16 (7–28)	17 (8–29)	12 (6–22)	

^a^4 missings

^b^1 missing

^c^168 missings

^d^Based on the vaccination first date in Spain

A total of 117 symptoms were collected, analysed by sex and period (Table S3, S4, S5) and subsequently gathered in 18 groups of symptoms to facilitate the analysis. Analysing the symptoms individually by time period, we found that the median number of symptoms per participant was 24 at baseline, 20 at 22–60 days and 16after 3 months, being higher in women at the three cut-offs than in men.

### Symptoms

**Fig. 2:**
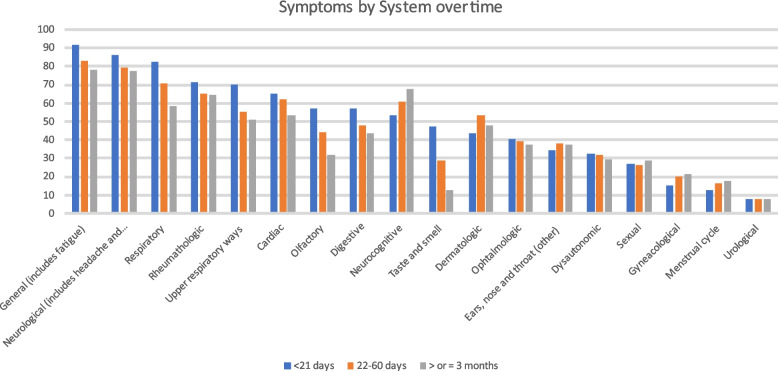
Evolution of symptoms grouped by system over time

As shown in Fig. 2, percentages of grouped symptoms are presented at baseline, 22–60 days and ≥ 3 months showing that most of the symptoms’ system frequency decreased over time, some remained almost the same, such as dermatological, disautonomic, urological and ENT, and others such as menstrual, sexual, gynaecological, and neurocognitive increased.

General (including tiredness or fatigue, dysthermia, fever, general malaise, inappetence, weight loss, muscle pain, oral herpes) and neurologic symptoms were the most frequently reported by all respondents at all time cut-off points.

**Fig. 3:**
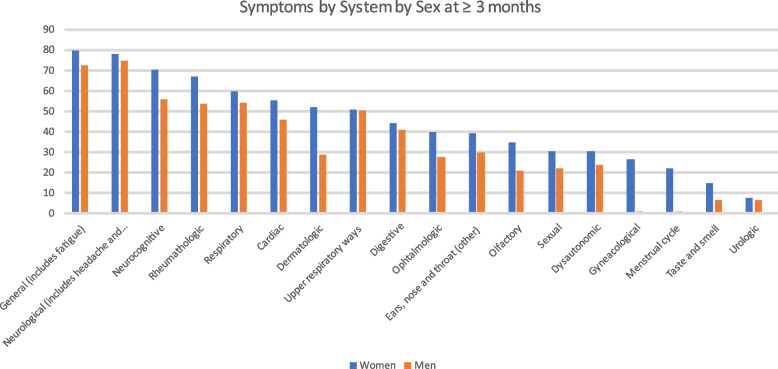
Symptoms by system by sex at ≥3 months

By sex, at baseline the most frequent groups of symptoms in both sexes were the general (92.8% in women, 87.2% in men), followed by the neurologic ones in women (88%) and the respiratory (79%) ones in men. The big difference observed between sexes at all cut-offs was in dermatologic symptoms followed by olfactory symptoms, both of which were more frequent in women than in men (Table S6). The evolution of symptoms by system is shown in Fig. 3.

By age, we found was that olfactory symptoms were widely reported at baseline for the 18–34 years group (68.5%), more than at any other age and that respondents aged 50–64 years old reported a major frequency of respiratory symptoms (83.5%) than other ages respondents). The most frequent symptoms reported at ≥3 months at age 50–64 were neurological (81.5%), while the most common symptoms in other age groups were the general ones. A significant finding is that the frequency of general symptoms at the three cut offs points was lower in those aged + 64 years than in any other age range (68.1%) (Table S7).

By wave, general symptoms were the most reported for the three waves at the three-time cut-off points for baseline, 22–60 days and ≥ 3 months, while neurocognitive symptoms increased their prevalence among the first and second waves in the three-time cut-off points. Olfactory symptoms were more frequent in the second (58.9%) and third (63.4%) waves in the first 21 days from symptom onset and their prevalence decreased by more than 10% over time in all waves at ≥3 months (Table S8).

We analysed symptoms by microbiological diagnostic testing and found no significant differences in symptoms between participants who had a positive RAT or PCR and those who did not, except for olfactory alterations that were more common during the first 21 days in those who had a positive test (63.6%) than in those who did not (50.8%), taste and smell alterations (53.9% of those who had a positive test and 39.3% of those wo hadn’t) (Table S9).

**Fig. 4:**
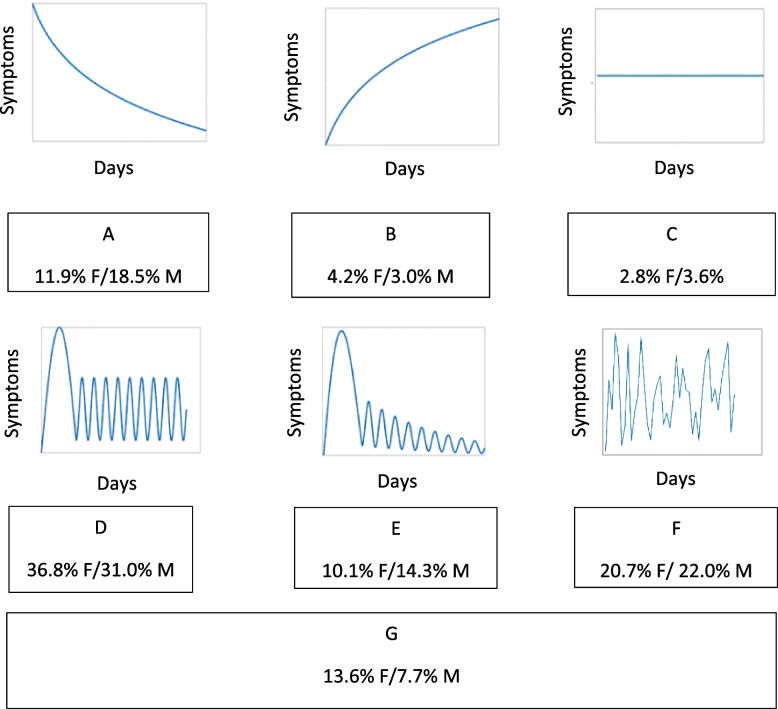
Representation of the perceptions of participants on their symptom’s evolution during time. F = female; M = men. Percentages refers to the frequency of each graphic in each sex. **A** Symptoms were very intense at first 3–4 weeks and progressively decrease.; **B** Symptoms increase their intensity for the first 3–4 weeks and haven’t decrease its intensity.; **C** Symptoms have maintained same intensity from the beginning since nowadays; **D** Symptoms were of high intensity for the first 3–4 weeks and then persist, intensifying, in a cyclical way, without disappearing completely; **E** Symptoms were of high intensity for the first 3–4 weeks and after that, decreased their intensity fluctuating, until they disappear; **F** Symptoms intensity don’t follow any pattern that I can identify; **G** No graphic represents my perception of my symptom’s evolution over time

The self-reported symptom evolution of participants was included in the questionnaire. Figure 4 shows the representation of the self-perceptions of participants on symptom evolution over time. For both sexes and at all ages, the most frequent evolution was “Symptoms were of high intensity for the first 3-4 weeks and then persist, intensifying, in a cyclical way without disappearing completely” (36.8% in women and 31% in men) (Fig. 4D). The second most frequent evolution was the one with no identified pattern (20.7% in women and 22.0% in men) by people affected at any age (Fig. 4F), except for the 50–64 age group where the second most frequent evolution was high symptom intensity followed by a progressive decrease in their intensity until disappearance (Fig. 4E).

### Clusters of symptoms

**Fig. 5:**
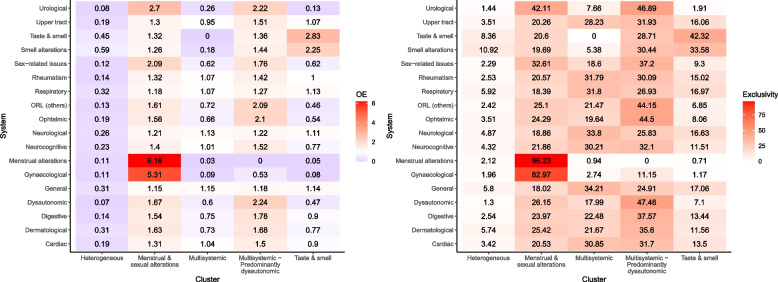
Groups of symptoms included in each cluster by OE and Exclusivity

Five clusters were identified and named according to the systems most predominantly affected based on the OE ratio and exclusivity of each cluster (Fig. 5): *Multisystemic, Multisystemic – predominantly dysautonomous, Heterogeneous, Taste & smell,* and *Menstrual & sexual alterations.*

The explained variance and the loadings of the PCAMix transformation can be found at supplementary data, Fig. S3.

**Fig. 6:**
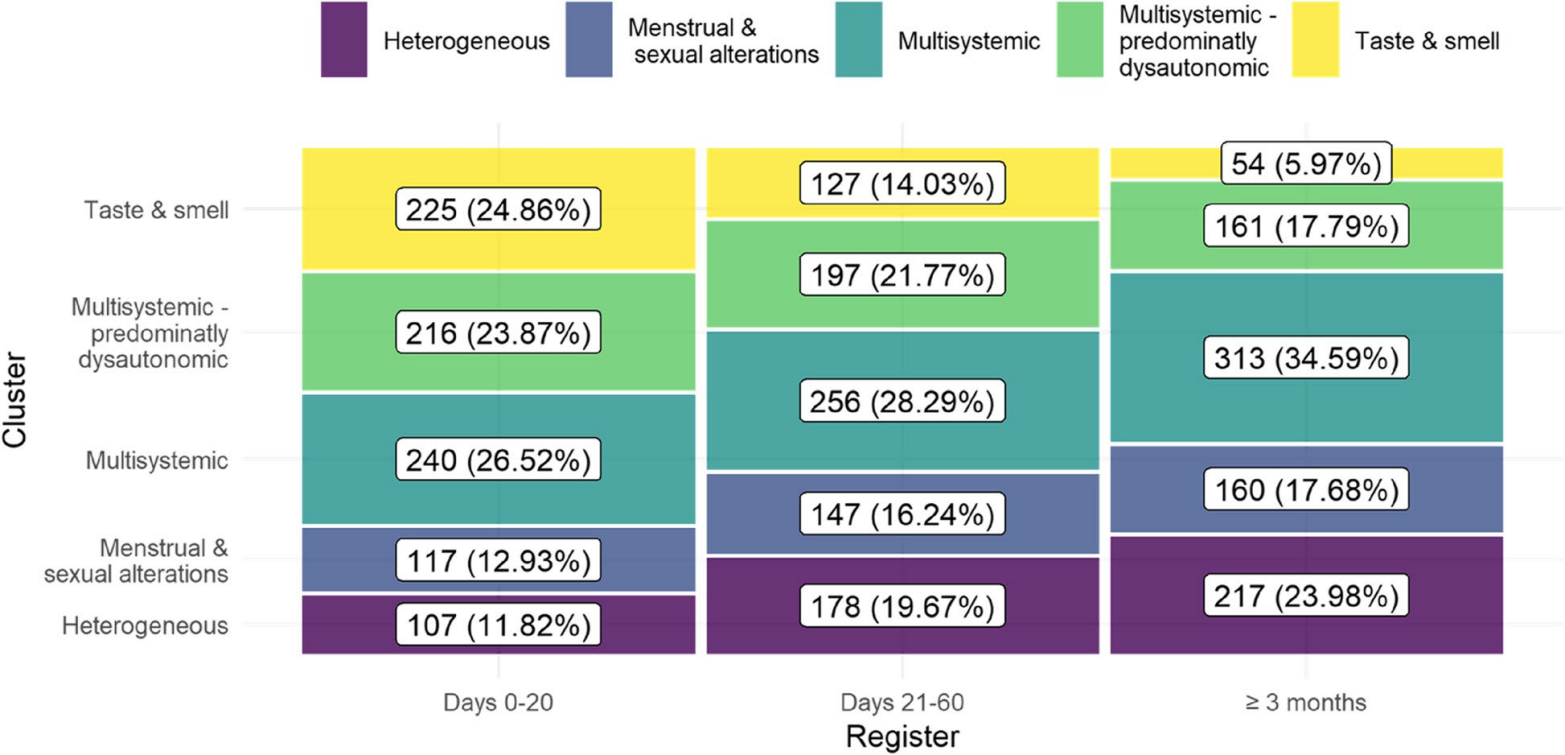
Prevalence of clusters over time. The percentage reports the prevalence on each time period

*Multisystemic* and *Multisystemic – predominantly dysautonomic* were the most common clusters, gathering 29.8 and 21.1% of the records during the follow-up period, respectively. *Heterogeneous*, a cluster in which no single system is predominantly affected, gathered 18.5% of the records. It was followed by *Menstrual & sexual alterations* (15.6%), and *Taste & smell* (15.0%). *Taste & smell* and *Multisystemic* were the most common clusters at the beginning of the condition, while *Heterogeneous* and *Multisystemic* were more common after 3 months (see Fig. 6). The prevalence of all clusters except *Taste & smell* and Multisystemic *– predominantly dysautonomic* increased over time (see Fig. 6).

**Fig. 7:**
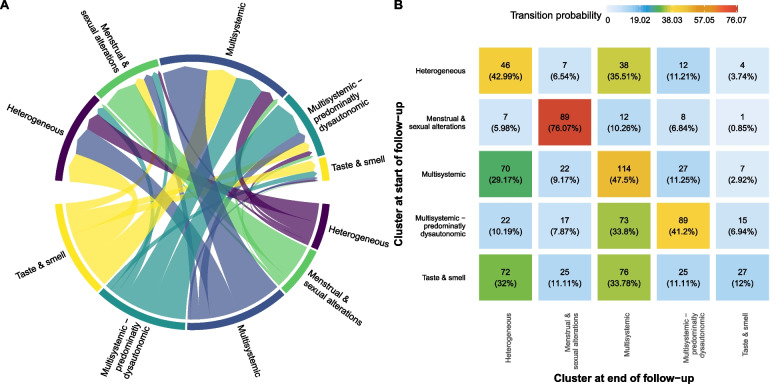
Transitions and cluster evolution over time. **A** shows the transitions from the cluster at the start of the follow-up (bottom) to the cluster at the end of follow-up (top). **B** shows the transition matrix of these transitions, reporting the percentage of individuals that changed from one initial cluster (rows) to a final cluster (columns)

Some clusters were more stable over time than others. For example, 76.1% of participants who started with *Menstrual & sexual alterations* remained in this same cluster > 60 days, while only 12% of participants in *Taste & smell* stayed in it and 32 and 33.8% of them changed to *Heterogeneous* and Multis*ystemic*, respectively. Participants gathered in *Multisystemic* mainly either remained in the same cluster (47.5%) or transitioned to *Heterogeneous (*29.2%). Similarly, participants with *Multisystemic – predominantly dysautonomic* affection mostly either transitioned to *Multisystemic* (33.8%) or remained in the same cluster (41.2%), while participants with a *Heterogeneous* affection either remained in it (43%) or transitioned to *Multisystemic (35.1%)* (see Fig. 7).

## Discussion

This study presents the evolution of persistent COVID-19 symptoms at three-time cut-off points in a cohort of 905 people in Catalonia. The key findings are as follows: 1) The pattern of symptom evolution observed at the three cut-off points (baseline, 22–60 days and ≥ 3 months) was a decrease in the frequency of many of the symptoms (digestive, upper respiratory tract, olfactory, ophthalmologic, respiratory, cardiac, rheumatologic, general, neurologic, disautonomic and taste and smell). 2) Neurocognitive, dermatological, ENT symptoms, gynaecological, sexual menstrual symptoms increased. 3) Urologic symptoms remained stable. 4) The most frequent clusters at baseline were *Taste & smell* and *Multisystemic*. 5) The most frequent cluster at ≥3 months was *Multisystemic*. We have examined the progression of COVID-19 symptoms towards long COVID-19, enabling the execution of a pertinent clinical investigation for the management of individuals in care and providing insights into the clinical course of long COVID-19.

The data are similar to other studies reviewed. They show a predominance of women younger than men, who had more comorbidities, the most frequent being allergy, and report no previous treatments [9, 21, 44–47]. However, the women interviewees did not smoke and had an average “normal weight” BMI. These last two characteristics differ from those reported by other researchers [2, 21, 22].

Most of the women in our cohort caught the disease during the first wave (60.1%) and had a positive diagnostic test (PCR or RAT) at some point in its course (52.4%).

Beyond 3 months of symptom onset, respondents reported a mean of 16 symptoms with a higher number of symptoms in women (17 symptoms) than in men (12 symptoms). This is similar to data from other studies which reported means of 13.76 and 55.9 symptoms per patient [2, 21, 48].

Some studies suggest that greater involvement in women may be related to a different expression of angiotensin converting enzyme 2 (ACE-2) or transmembrane protease serine 2 (TMPRSS2) receptors or to lower production of proinflammatory cytokines such as interleukin-6 (IL-6) in women after a viral infection [49]. However, the sex difference in our cohort might be due to greater involvement of women than men. It is known that women may be more able to express symptoms or allow themselves to express them more than men, whereas men are more restricted in expressing symptoms in order to conform to hegemonic masculinity patterns [50–54]. We also consider that the higher frequency of women’s participation in this study may have to do with the fact that women tend to look after their health more, as has been described in a number of studies [55]. The higher frequency of symptoms which are more difficult to refer for consultation, such as fatigue or brain fog, may mean that they are underestimated, especially in women (gender bias) when treating women with persistent symptoms which would not be found when treating a man reporting the same symptoms.

General symptoms predominated in our cohort in both sexes in the first 21 days and in the cut off 22–60 days. Neurocognitive symptoms were more common in women. These results are similar to those reported in studies conducted in other countries [46, 56]. After 3 months, general symptoms were the most frequent symptoms in women and neurological in men, but neurological symptoms were the seconds in frequency reported by women, most likely related to continued headache. These results are close to the ones found by Ballering et al., who describes as a core Long COVID symptoms those that in our cluster analysis will correspond to Multisystemic cluster and Multisystemic-predominantly disautonomic cluster [57]. Neurocognitive symptoms were predominant, especially in the 35–49 and in the 50–64 year ages groups, along with general and neurologic symptoms, which is consistent with the studies reviewed [2, 21, 26, 44, 56, 58]. Furthermore, differences between men and women in the frequency of dermatological symptoms are striking across all time cut-off points in the study where they are more frequent in women. Some researchers point to a potential relationship between dermatological symptoms and systemic inflammation and between systemic inflammation and neurocognitive symptoms [59]. Olfactory symptoms were also more present in women than in men and persisted more over time in this group as reported in published meta-analyses [60, 61].

Most of our cohort was infected in the first and second waves. It is noticeable that the frequency of olfactory symptoms during the first 21 days increased in the second and third waves compared to the first. A study following a cohort of individuals who experienced COVID-19 in Norway indicates that 16.6% of those infected during the first wave still had olfactory- and taste-related symptoms 1 year later [62]. Another study [27] including anosmia and dysosmia as part of the central neurological cluster indicated that this neurological cluster was the largest cluster in both the alpha and delta variants [27].

From a clinical point of view, it is important to know which clusters may be found in the acute phase of SARS-COV-2 infection and which patterns those initial symptoms and clusters follow over a number of time cut-off points while they persist. This can enable health professionals to better suspect and identify a Long COVID condition in clinical appointments by symptoms and cluster evolution at different moments in time. Learning about cluster trends might also help health systems to improve their delivery of care to Long COVID patients [63].

The clusters defined in our study are justified for two different reasons. Firstly, a mathematical validation to choose the clustering hyperparameters was performed: The number of clusters (from 2 to 8) and degree of fuzziness (from 1.1 to 1.8, per 0.1) was validated were chosen through by validation indices calculated 100 times in order to account for the random nature of the clustering initialisation. In addition, the most determinant conditions on each cluster were selected through the OE and the exclusivity. Secondly, the mechanisms by which long COVID-19 manifests are multiple, complex, and often overlap. The clusters obtained, such as the multisystemic one, are conditioned by various pathophysiological mechanisms, including Mast Cell Activation Syndrome), Myalgic Encephalomyelitis/Chronic Fatigue Syndrome, and Postural Orthostatic Tachycardia Syndrome. These are justified in the different clusters observed in this paper [63]. In our data, the most prevalent clusters observed were Multisystemic and Multisystemic-predominantly disautonomic. We noted that these clusters stabilised over time with either the second becoming part of the former or the former becoming part of the Heterogeneous group. Furthermore, the transitions over time of clusters might suggest a tendency towards unspecificity or heterogeneity of symptoms that could point to an improvement in symptoms or greater adaptation of people to the symptoms after a long period of experiencing them. Kenny et al. report that the most heterogeneous of the three clusters they found is the one that includes the most people and suggest that this heterogeneity may be a sign of recovery [26]. Contrary to our results, Whitaker et al. [64] identify two stable clusters over time, one of which includes fatigue, shortness of breath and chest pain or tightness and the other with a high prevalence of smell and taste disturbances [64]. Cluster changes over time underscore Long COVID’s multisystemic nature. Data analysed using cluster methodology indicate that there is no specific timeline for recovery from long COVID, as it appears to depend on individual risk factors, including psychological factors, and the severity and spectrum of symptoms experienced. Some studies indicate that the total time to complete symptom resolution reported in the literature for patients with long COVID is highly variable, with the average time to symptom resolution being 4 months in non-hospitalized patients and 9 months in those with more serious cases [29, 65, 66].

The menstrual cluster and menstrual symptoms increased across the three cut-off points probably because over time there are more cycles to assess the disturbance. Most of the reviewed studies on persistent COVID that feature clusters do not include symptoms relating to the menstrual cycle [21, 26, 44, 61, 67–69]. Those that did consider them found changes in the volume and duration of the cycle; some saw them as part of a heterogeneous group of genitourinary symptoms, where 62.5% of respondents reported disorders, while others included them in a group of gynaecological disorders which remained stable over time [2, 70, 71]. We included menstrual symptoms in our study at the request of the group of people affected and because the rest of the research team was concerned that this information was often downplayed in the medical setting. It also speaks to the need to make menstrual health visible and relevant to women’s health research as a public health issue and also as a matter of human rights [72].

Several studies examine the evolution and transitions over time of clusters, yet there are no common clusters across studies [2, 21, 26, 44, 64, 68, 73, 74]. Between-study differences are due to the varying symptom classification, the analysis techniques used, and the number of people included in each study that shape the symptom clusters identified. These differences are also a result of the time at which symptoms are identified in relation to the initial disease [2, 18, 23, 37, 51–54]. This heterogeneity hampers comparison between studies.

Thus, the evolution and transitions of long COVID-19 symptom clusters over time are complex and variable, with different trajectories and phenotypes being identified. Further research is needed to better understand the long-term implications of these symptoms and to guide monitoring and treatment strategies for individuals with long COVID-19.

### Strengths and limitations

The study’s strengths include the fact that it is co-created and stems from a commitment made to the people in the Long COVID-19 group in Catalonia. The analyses have been differentiated by sex, whereas few studies have stratified persistent COVID results by sex [75]. Moreover, this is a longitudinal study that involves cluster analysis. The inclusion of menstrual symptoms is not described in many publications on persistent COVID and is one of this study’s strengths.

Compared with hierarchical clustering, fuzzy c-means cluster analysis is less susceptible to outliers in the data, choice of distance measure and the inclusion of inappropriate or irrelevant variables [76]. Nevertheless, some disadvantages of the method are that there may be different solutions for each set of seed points and there is no guarantee of optimal clustering [77]. To minimise this shortcoming, we carried out 100 cluster realisations with different seed points to use the average result of all of them. In addition, although the method is not efficient when a large number of potential cluster solutions are to be considered, this was not the case of our study [76].

However, this study is not without limitations. Not least of them is the likelihood of recall bias since recruitment began in December 2020 and we also included individuals already infected in the first wave and therefore with retrospective data in this subgroup. The fact that this is a self-reported survey may be a limitation for some, although we think it values the experience of the affected person as a source of knowledge in addition to how a professional might subjectively assess an affected person’s narrative.

The individuals included in the study were part of the social networks of activists, close people or contacts of contacts. We are aware that we have not been able to access all people with long COVID and that can introduce a selection bias. At the time of data collection this was a possible and feasible way. Two reasons account for this: 1) the limited number of face-to-face meetings due to outbreak restrictions 2) the limitation due to the physical conditions of the participants. Our sampling was performed by convenience and snowball sampling, with the advantages and disadvantages of this sampling strategy.

The inclusion of people with an end date of symptoms in the main analysis could lead to a bias, but two things might be of consideration. On one hand, these people had more than 3 months of symptom evolution so, they were labelled as Long COVID. On the other hand, as there is no definition for “recovery” (relapses being a common evolution of the condition), we consider it was better to include them and follow them up in the second phase of the study to see if they relapsed or not.

At the beginning of the pandemics, the lack of tests for non-hospitalised patients made it hard to confirm a SARS-COV-2 infection. Although the inclusion of people who never tested positive for SARS-COV-2 could be seen as a limitation, we see it as a matter of justice to people affected who had no access to the test.

The gender imbalance can introduce biases and limit the generalizability of the study findings, as the experience of men with Long COVID may not be accurately reflected due to the lower number of men.

We are aware that the selection of sex, age and systems as variables and no other variables such as comorbidities or disease severity provides one perspective of understanding Long COVID from multiple perspectives existing, such as the quality and relevance of the results are highly dependent on the input variables chosen by the analysis.

The respondents were probably not representative of people with persistent COVID as most of them were members of the Long COVID-19 group in Catalonia, albeit the description of the characteristics of this group is also one of our study’s strengths. There may thus be a selection bias in the fact that many of the participants were recruited by the Long COVID-19 group in Catalonia and were more willing to participate in a study about their condition. So, replication of the study using different datasets and populations could be necessary to assess the generalizability of the results.

Not having a control group of non-infected participants could alter the validation of the finding.

Vaccination status and reinfection were not considered in our questionnaire. Recruitment started before the announcement of the vaccination programme (which started on 27th December 2020) in Spain. Vaccinated status and reinfection might be confounding factors when assessing the frequency of symptoms in those who reported symptom onset in 2021 [78, 79].

## Conclusions

People with persistent COVID in our cohort reported general and neurological symptoms as the most frequent initial symptoms followed by respiratory symptoms in both women and men. Over time, neurocognitive symptoms displaced respiratory symptoms in women, while respiratory symptoms remained the third most frequent symptom group in men. The greatest differences between sex were found in dermatological and olfactory symptoms which were more frequent in women at all time cut-off points. In cluster analysis, evolution towards a more heterogeneous cluster over time might suggest stabilisation of the disease or adaptation to the symptoms. Heterogeneity of symptoms may render the clinical picture vague and indeterminate. This, coupled with potential gender bias, restricted access to diagnostic testing during the first wave and the change in current Spanish protocols for screening for SARS-COV-2 infection, may interfere with and hinder recognition of and care for people with persistent symptoms.

### Supplementary Information

**Additional file 1: Figure S1.** How symptoms evolution graphics were constructed.

**Additional file 2: Figure S2.** Graphs representing T-Trend of each group of symptoms. A= Menstrual; B= Olfactory; C= Cardiologic; D= Dermatologic; E= Digestive; F= Disautonomic; G= Sexual; H= General; I= Gyneacological; J= Neurocognitive; K= Neurologic; L= Ophtalmologic; M= Taste and Smell; N= Ear, Nose and Throath; O= Respiratory; P= Rheumatic; Q=Urologic; R=Upper Respiratory Ways.

**Additional file 3: Figure S3.** Visualization of the records on the first two PCAmix dimensions, coloured by cluster (A), and visualization of the squared loadings (magnitude and direction of the coefficients for the original variables) (B).

**Additional file 4: Table S1.** Symptoms classification by system.

**Additional file 5: Table S1.** Symptoms groups overtime by sex with t-Trend.

**Additional file 6: Table S2.** Characteristics of end-date of symptoms cohort.

**Additional file 7: Table S3.** Symptoms by sex at baseline.

**Additional file 8: Table S4.** Symptoms by sex at 22-60 days.

**Additional file 9: Table S5.** Symptoms by sex at ≥ 3 months.

**Additional file 10: Table S6.** Symptoms by system by sex overtime.

**Additional file 11: Table S7.** Symptoms by system by age.

**Additional file 12: Table S8.** Symptoms by system by wave over time.

**Additional file 13: Table S9.** Symptoms by system by PCR or RAT result.

## Data Availability

In accordance with current European and national law, the data used in this study are only available for the researchers participating in this project. Thus, we are not allowed to distribute the data or make them publicly available to other parties. The original REDCap questionnaire will be available under request. For further information, contact the corresponding author.

## Abbreviations

SARS-COV-2: Severe Acute Respiratory Syndrome Coronavirus 2
COVID-19: coronavirus disease
ICU: Intensive Care Unit
PCR: Polymerase Chain Reaction
RAT: Rapid Antigen Test
WHO: World Health Organisation
EHR: Emergency Health Room
IDIAP Jordi Gol: Fundació de Recerca en Atenció Primària de Salut Jordi Gol
REDCap: Research Electronic Data Capture
RENAVE: Red Nacional de Vigilancia Epidemiológica
PCAMix: Principal Component Analysis of Mixed Data
BMI: Body Mass Index
ENT: Ear, nose, throat
ACE-2: Angiotensin Converting Enzyme 2
TMPRSS-2: Transmembrane protease serine 2
IL-6: Interleukin 6
