# Supplementary figures and images for: Characterisation, symptom pattern and symptom clusters from a retrospective cohort of Long COVID patients in primary care in Catalonia
Source: BMC Infect Dis. 2024 Jan 15;24:82. doi: 10.1186/s12879-023-08954-x (PMC10789045; doi:10.1186/s12879-023-08954-x)

**FIGURE S1. How symptoms evolution graphics were constructed.**


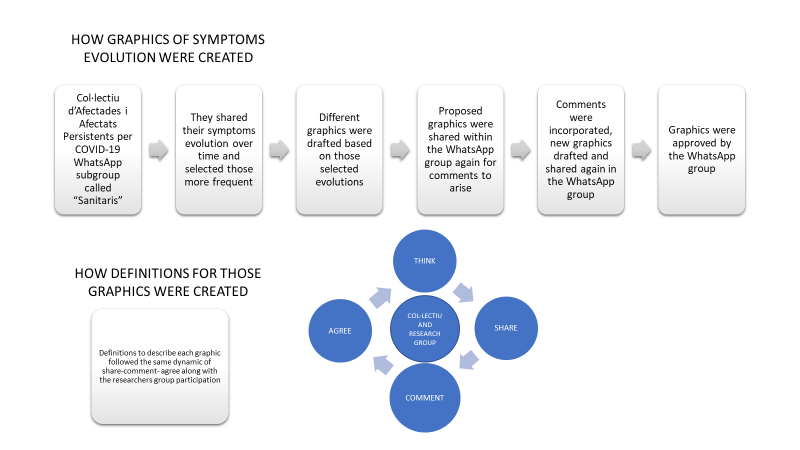

Supplement: Supplementary file 1 — Additional file 1: Figure S1. How symptoms evolution graphics were constructed. [file 12879_2023_8954_MOESM1_ESM.docx]
