# Supplementary material for: Characterisation, symptom pattern and symptom clusters from a retrospective cohort of Long COVID patients in primary care in Catalonia
Source: BMC Infect Dis. 2024 Jan 15;24:82. doi: 10.1186/s12879-023-08954-x (PMC10789045; doi:10.1186/s12879-023-08954-x)
Supplement: Supplementary file 2 — Additional file 2: Figure S2. Graphs representing T-Trend of each group of symptoms. A= Menstrual; B= Olfactory; C= Cardiologic; D= Dermatologic; E= Digestive; F= Disautonomic; G= Sexual; H= General; I= Gyneacological; J= Neurocognitive; K= Neurologic; L= Ophtalmologic; M= Taste and Smell; N= Ear, Nose and Throath; O= Respiratory; P= Rheumatic; Q=Urologic; R=Upper Respiratory Ways. [file 12879_2023_8954_MOESM2_ESM.docx]

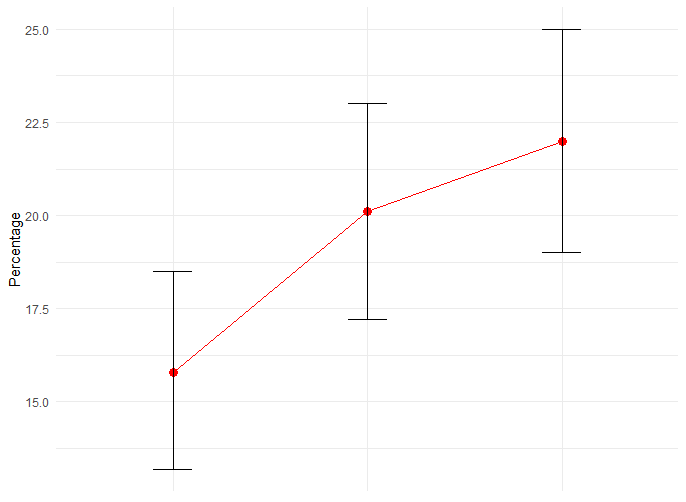


**A**


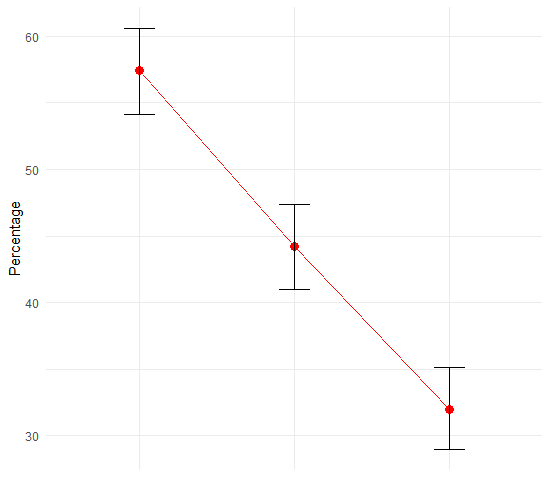

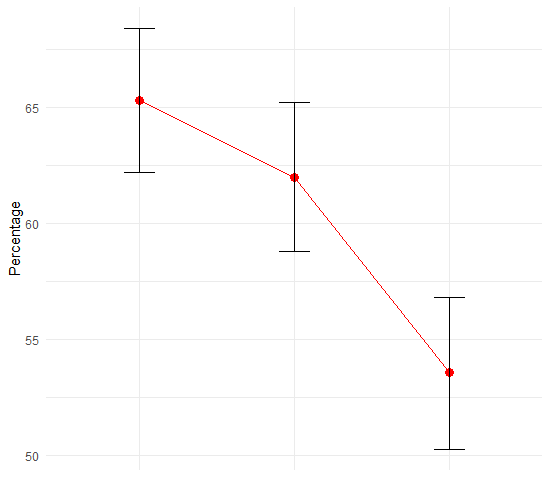

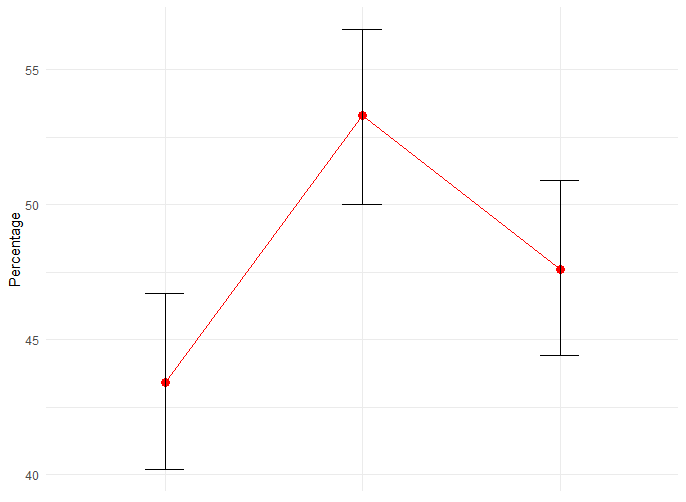

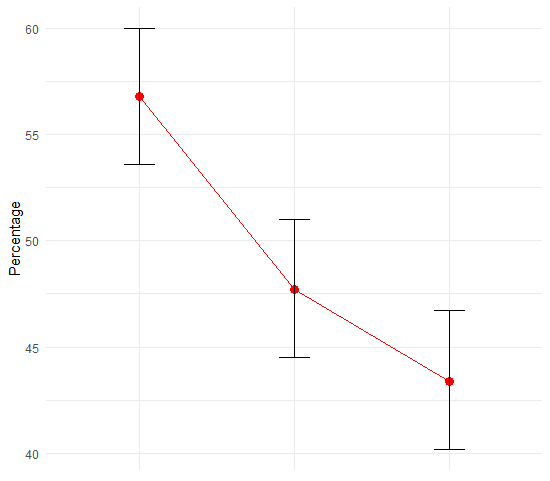

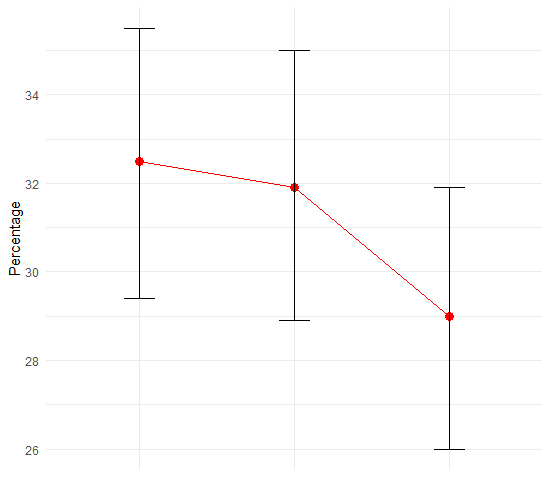

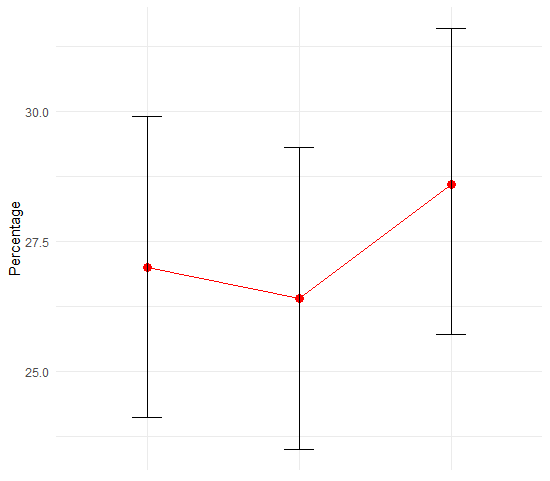

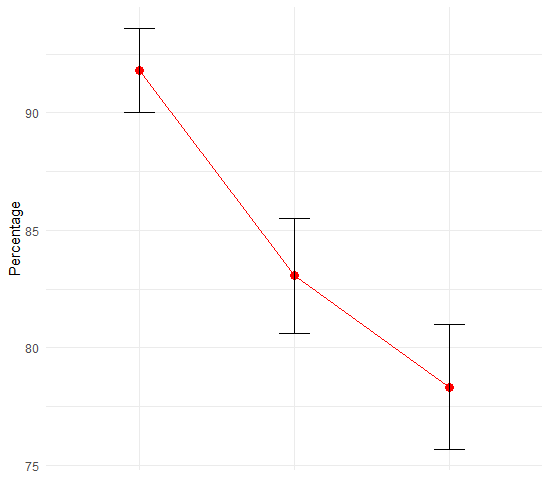

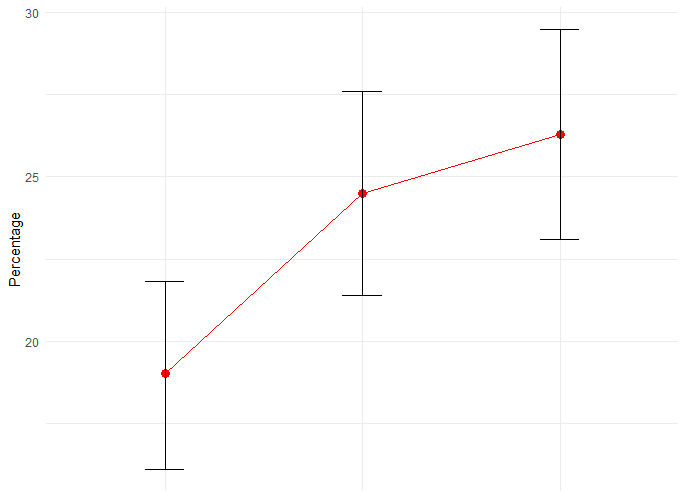

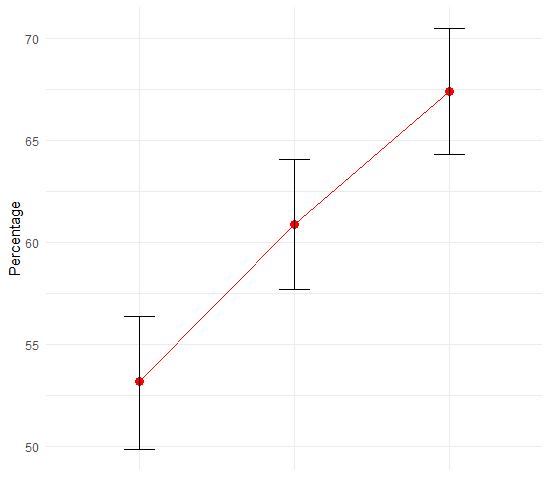

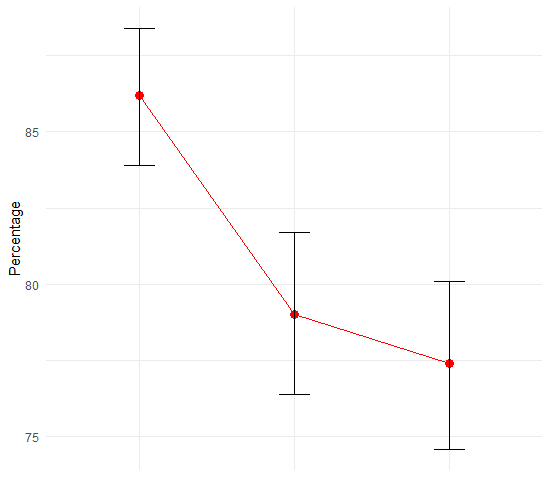

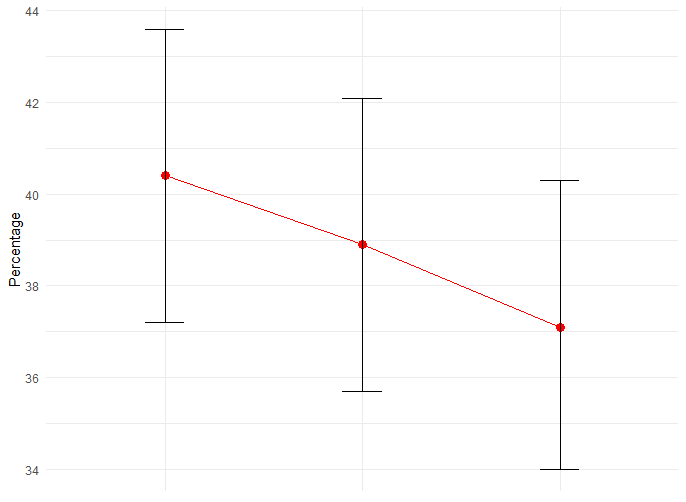

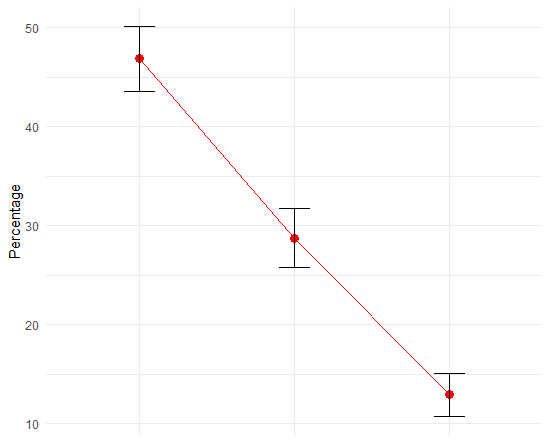

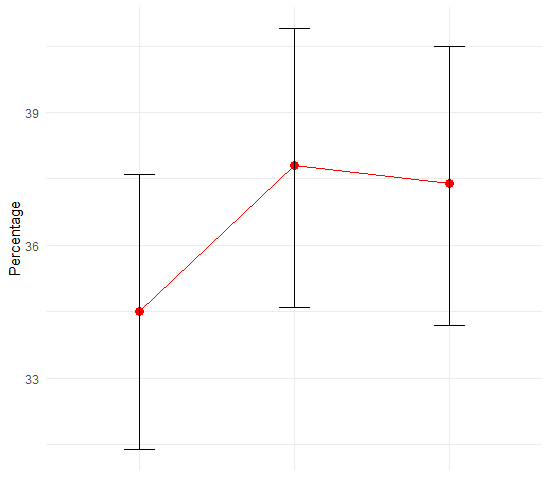

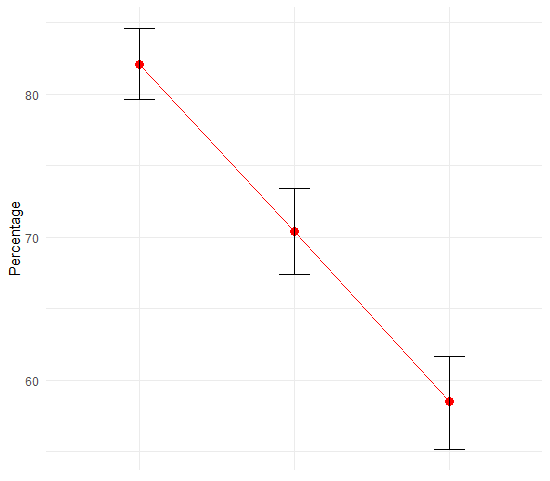

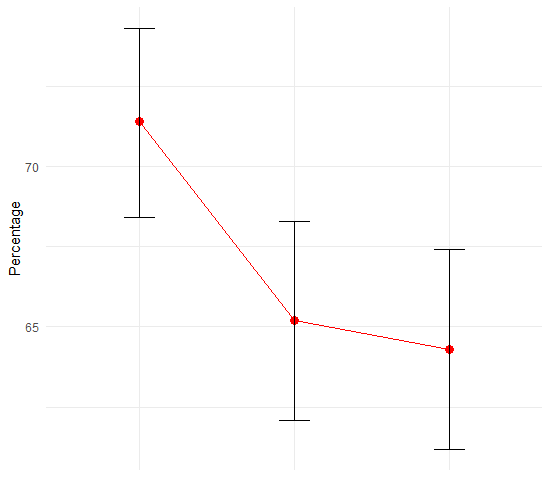

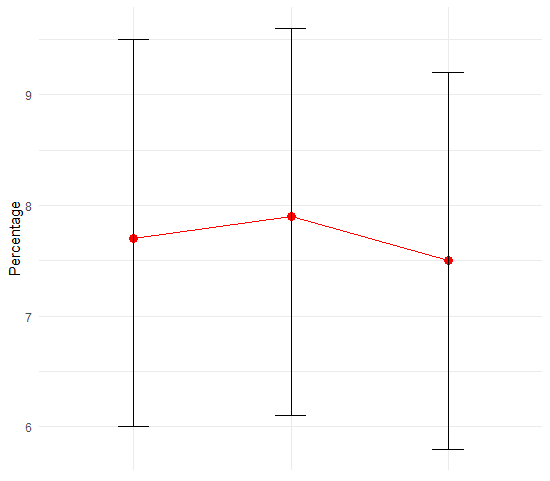

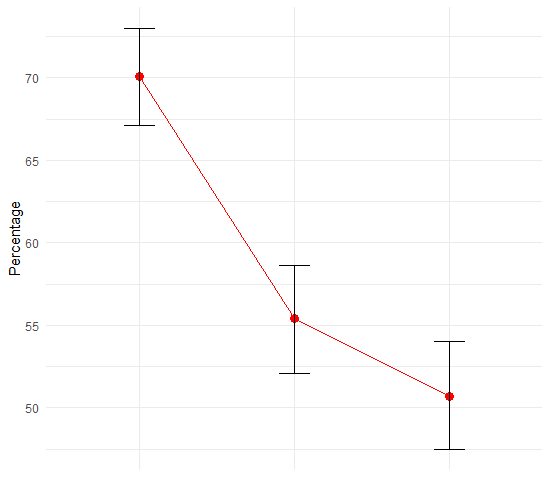


**B**

**C**

**D**

**E**

**F**

**G**

**HH**

**I**

**J**

**K**

**L**

**M**

**N**

**O**

**P**

**Q**

**R**

**Figure S2**. Graphs representing T-Trend of each group of symptoms. A= Menstrual; B= Olfactory; C= Cardiologic; D= Dermatologic; E= Digestive; F= Disautonomic; G= Sexual; H= General; I= Gyneacological; J= Neurocognitive; K= Neurologic; L= Ophtalmologic; M= Taste and Smell; N= Ear, Nose and Throath; O= Respiratory; P= Rheumatic; Q=Urologic; R=Upper Respiratory Ways.
