# Supplementary material for: Characterisation, symptom pattern and symptom clusters from a retrospective cohort of Long COVID patients in primary care in Catalonia
Source: BMC Infect Dis. 2024 Jan 15;24:82. doi: 10.1186/s12879-023-08954-x (PMC10789045; doi:10.1186/s12879-023-08954-x)
Supplement: Supplementary file 3 — Additional file 3: Figure S3. Visualization of the records on the first two PCAmix dimensions, coloured by cluster (A), and visualization of the squared loadings (magnitude and direction of the coefficients for the original variables) (B). [file 12879_2023_8954_MOESM3_ESM.docx]

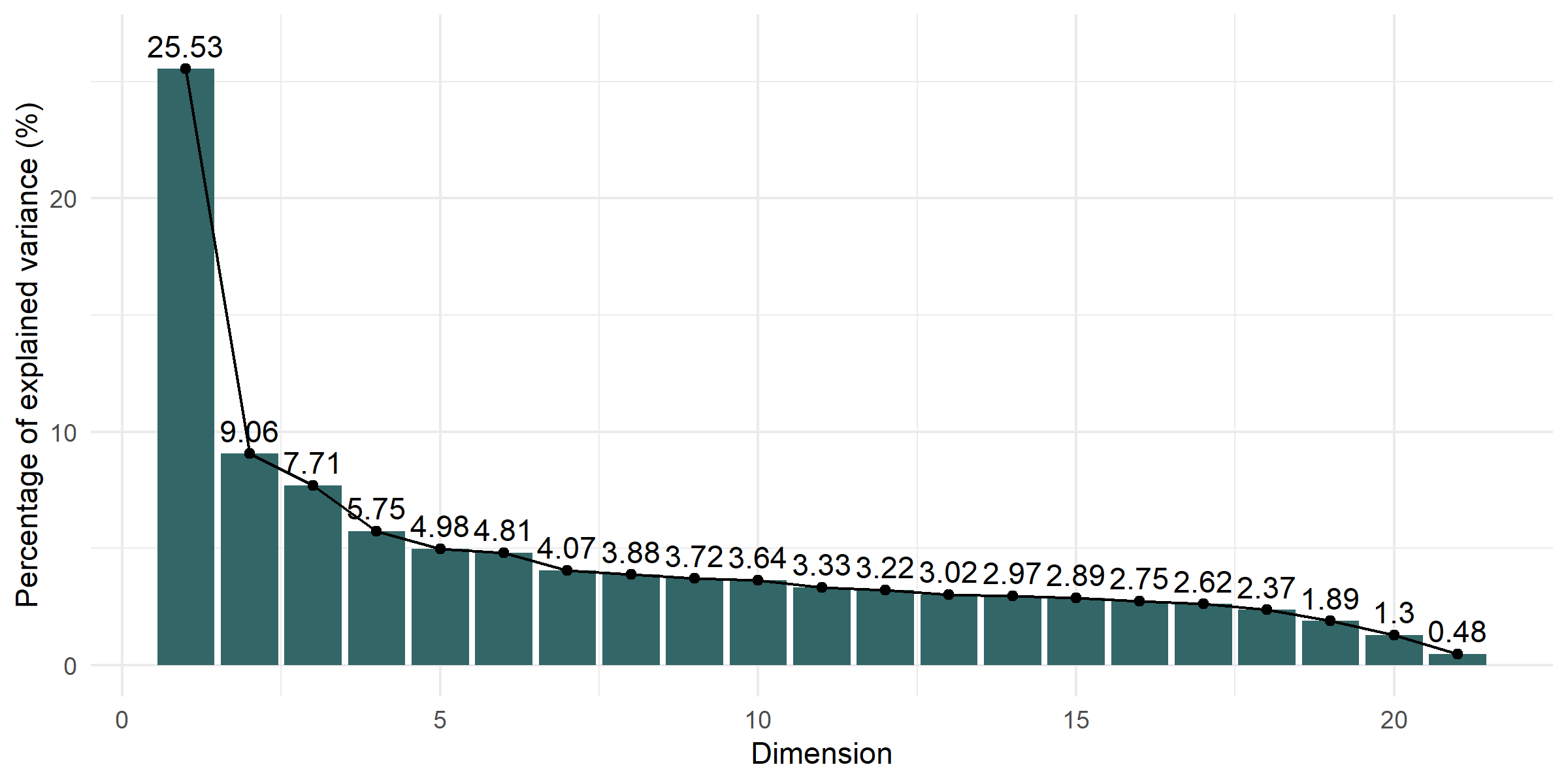


Figure 1. Percentage of explained variance for the PCAMix dimensions


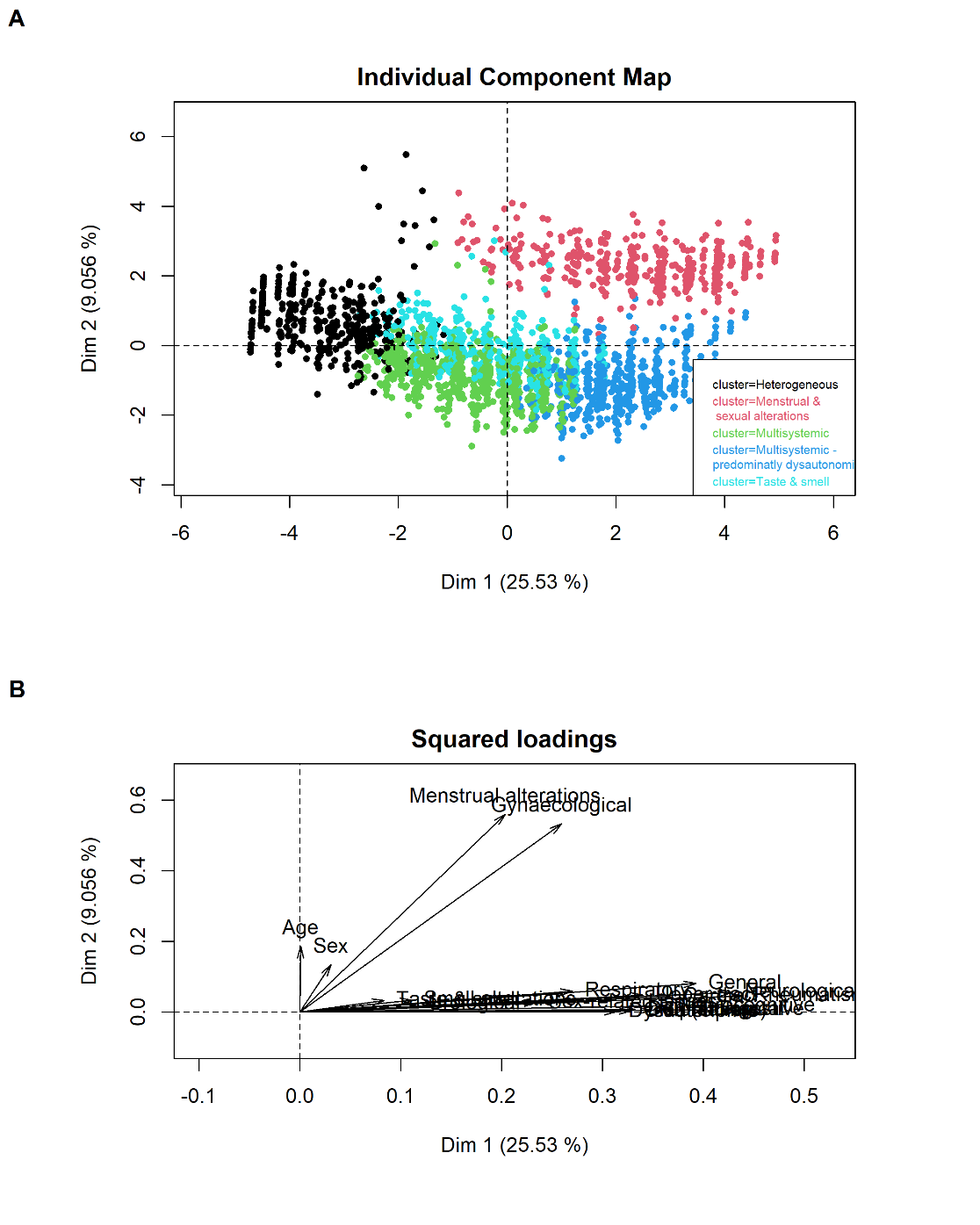


Figure S3. Visualization of the records on the first two PCAmix dimensions, coloured by cluster (A), and visualization of the squared loadings (magnitude and direction of the coefficients for the original variables) (B).
