# Supplementary material for: Characterisation, symptom pattern and symptom clusters from a retrospective cohort of Long COVID patients in primary care in Catalonia
Source: BMC Infect Dis. 2024 Jan 15;24:82. doi: 10.1186/s12879-023-08954-x (PMC10789045; doi:10.1186/s12879-023-08954-x)
Supplement: Supplementary file 4 — Additional file 4: Table S1. Symptoms classification by system. [file 12879_2023_8954_MOESM4_ESM.docx]

**TABLE S1: Symptoms classification by system**

**RESPIRATORY SYMPTOMS**

- Productive cough
- Dry cough
- Shortness of breath at
  - Moderated efforts
  - Small efforts
  - Rest
- Esputum
- Incomplete inspiration
- Abnormal breathing
- Low oxygen saturation
- Blue lips

**CARDIAC SYMPTOMS**

- Palpitations
- Chest pain
- Oppressive chest pain
- Burning chest pain
- Other kind of chest pain
- Tachycardia
- Bradycardia
- Hypotension
- Hypertension

**NEUROLOGIC SYMPTOMS**

- Headache
- Dizziness
- Tingling
- Muscle weakness
- Trembling
- Convulsions
- Hypoesthesia
- Cramps
- Fasciculations
- Incoordination
- Difficulty in fine motor skills
- Onset insomnia
- Maintenance insomnia

**NEUROCOGNITIVE**

- Lack of concentration
- Oversights
- Difficulty in remembering.
- Disorientation
- Anomie
- Alexia

**DISAUTONOMIC SYMPTOMS**

- Inappropriate perspiration
- Frequent micturition

**TASTE AND SMELL**

- Loss of taste
- Loss of smell
- Cacosmia
- Phantosmia

**OLFACTORY SYMPTOMS**

- Loss of smell
- Cacosmia
- Phantosmia

**EAR-NOSE-THROAT SYMPTOMS**

- Loss of hearing
- Excessive hearing
- Sore mouth
- Dry mouth
- Taste of blood on your throat
- Tinnitus
- Earache
- Dry nose
- Nosebleed (Epistaxis)
- Scrape in your throat
- Dysphonia
- Aphonia.
- Sonophobia

**UPPER RESPIRATORY WAY**

- Itchy nose
- Sneezing
- Mucus in the nose (rhinorrhoea)
- Nasal congestion
- Itching throat
- Sore throat

**GENERALS**

- Dysthermia
- Temperature <37º
- Temperature 37.1-37.5º
- Temperature 37.6-38
- Temperature >38º
- Shivers
- Weight loss
- Inappetence
- Muscle pain
- Tiredness/fatigue
- General malaise
- Oral herpes

**RHEUMATOLOGIC**

- Joint inflammation
- Neck pain
- Rib pain
- Back pain
- Right hypochondrium pain
- Left hypochondrium pain.
- Joint pain
- Pins and needles-like pain
- Pain from old injuries

**DERMATOLOGIC**

- Hair loss
- Dry Skin
- Itchy skin
- Rash on the skin
- Erythema pernio
- Cutis marmorata

**OPHTALMOLOGIC**

- Dry eyes
- Painful eyes
- Conjunctivitis
- Red eyes
- Blurred vision
- Diplopia
- Photophobia

**DIGESTIVE**

- Abdominal pain
- Stomach pain
- Nausea
- Vomit
- Stool mucus
- Blood in the stool
- Diarrhoea
- Liquid stools
- Flatulence
- Intestinal sounds

**GYNAECOLOGIC**

- Dysmenorrhea
- Vaginal discomfort

**MENSTRUAL CYCLE RELATED**

- Altered menstruation.
- Changes in the length of the cycle
- Changes in the volume of the cycle

**UROLOGIC**

- Genital discomfort
- Dysuria

**SEXUAL**

- Sexual dysfunction
- Low sexual desire
