# Supplementary material for: Characterisation, symptom pattern and symptom clusters from a retrospective cohort of Long COVID patients in primary care in Catalonia
Source: BMC Infect Dis. 2024 Jan 15;24:82. doi: 10.1186/s12879-023-08954-x (PMC10789045; doi:10.1186/s12879-023-08954-x)
Supplement: Supplementary file 5 — Additional file 5: Table S1. Symptoms groups overtime by sex with t-Trend. [file 12879_2023_8954_MOESM5_ESM.docx]

Table S1. Symptoms groups overtime by sex with t-Trend

TOTAL (n=905)

| Symptoms | Baseline (%) | 22 60d (%) | ≥3 months (%) | P-trend |
| --- | --- | --- | --- | --- |
| Dermatologic | 43.4% (40.2%-46.7%) | 53.3% (50.0%-56.5%) | 47.6% (44.4%-50.9%) | <0.001 |
| Ophtalmologic | 40.4% (37.2%-43.6%) | 38.9% (35.7%-42.1%) | 37.1% (34.0%-40.3%) | 0.109 |
| Gynaecological* | 19.0% (16.1%-21.8%) | 24.5% (21.4%-27.6%) | 26.3% (23.1%-29.5%) | <0.001 |
| Menstrual cycle* | 15.8% (13.2%-18.5%) | 20.1% (17.2%-23.0%) | 22.0% (19.0%-25.0%) | <0.001 |
| Urologic_ | 7.7% (6.0%-9.5%) | 7.9% (6.1%-9.6%) | 7.5% (5.8%-9.2%) | 0.939 |
| Sexual | 27.0% (24.1%-29.9%) | 26.4% (23.5%-29.3%) | 28.6% (25.7%-31.6%) | 0.121 |
| Digestive | 56.8% (53.6%-60.0%) | 47.7% (44.5%-51.0%) | 43.4% (40.2%-46.7%) | <0.001 |
| Upper Respiratory Ways | 70.1% (67.1%-73.0%) | 55.4% (52.1%-58.6%) | 50.7% (47.5%-54.0%) | <0.001 |
| Olfactory | 57.4% (54.1%-60.6%) | 44.2% (41.0%-47.4%) | 32.0% (29.0%-35.1%) | <0.001 |
| Ear, Nose, Throat (ENT) | 34.5% (31.4%-37.6%) | 37.8% (34.6%-40.9%) | 37.4% (34.2%-40.5%) | 0.067 |
| Respiratory | 82.1% (79.6%-84.6%) | 70.4% (67.4%-73.4%) | 58.5% (55.2%-61.7%) | <0.001 |
| Cardiac | 65.3% (62.2%-68.4%) | 62.0% (58.8%-65.2%) | 53.6% (50.3%-56.8%) | <0.001 |
| Rheumatologic | 71.4% (68.4%-74.3%) | 65.2% (62.1%-68.3%) | 64.3% (61.2%-67.4%) | <0.001 |
| General | 91.8% (90.0%-93.6%) | 83.1% (80.6%-85.5%) | 78.3% (75.7%-81.0%) | <0.001 |
| Neurological | 86.2% (83.9%-88.4%) | 79.0% (76.4%-81.7%) | 77.4% (74.6%-80.1%) | <0.001 |
| Neurocognitive | 53.2% (49.9%-56.4%) | 60.9% (57.7%-64.1%) | 67.4% (64.3%-70.5%) | <0.001 |
| Disautonomic | 32.5% (29.4%-35.5%) | 31.9% (28.9%-35.0%) | 29.0% (26.0%-31.9%) | 0.038 |
| Taste and Smell | 46.9% (43.6%-50.1%) | 28.7% (25.8%-31.7%) | 12.9% (10.7%-15.1%) | <0.001 |

*only in women

WOMEN (n=727)

| Symptoms | Baseline (%) | 22 60d (%) | ≥3 months (%) | P-trend |
| --- | --- | --- | --- | --- |
| Dermatologic | 48.0% (44.4%-51.6%) | 57.9% (54.3%-61.5%) | 52.1% (48.5%-55.8%) | <0.001 |
| Ophtalmologic | 42.5% (38.9%-46.1%) | 39.9% (36.3%-43.4%) | 39.5% (35.9%-43.0%) | 0.184 |
| Gynaecological | 19.0% (16.1%-21.8%) | 24.5% (21.4%-27.6%) | 26.3% (23.1%-29.5%) | <0.001 |
| Menstrual cycle | 15.8% (13.2%-18.5%) | 20.1% (17.2%-23.0%) | 22.0% (19.0%-25.0%) | <0.001 |
| Urologic_ | 7.4% (5.5%-9.3%) | 8.1% (6.1%-10.1%) | 7.7% (5.8%-9.6%) | 0.810 |
| Sexual | 26.8% (23.6%-30.0%) | 27.1% (23.9%-30.3%) | 30.1% (26.8%-33.5%) | 0.009 |
| Digestive | 58.2% (54.6%-61.8%) | 48.7% (45.1%-52.3%) | 44.0% (40.4%-47.6%) | <0.001 |
| Upper Respiratory Ways | 71.4% (68.4%-74.3%) | 54.9% (51.6%-58.1%) | 51.0% (47.8%-54.3%) | <0.001 |
| Olfactory | 60.5% (57.0%-64.1%) | 47.3% (43.7%-50.9%) | 34.9% (31.5%-38.4%) | <0.001 |
| Ear, Nose, Throat (ENT) | 35.5% (32.0%-39.0%) | 39.2% (35.7%-42.7%) | 39.3% (35.8%-42.9%) | 0.046 |
| Respiratory | 82.7% (79.9%-85.4%) | 70.0% (66.7%-73.3%) | 59.6% (56.0%-63.1%) | <0.001 |
| Cardiac | 68.0% (64.6%-71.3%) | 63.6% (60.1%-67.0%) | 55.3% (51.7%-58.9%) | <0.001 |
| Rheumatologic | 73.9% (70.7%-77.1%) | 66.4% (63%-69.9%) | 66.7% (63.3%-70.1%) | <0.001 |
| General | 92.9% (91%-94.7%) | 83.1% (80.4%-85.8%) | 79.6% (76.7%-82.6%) | <0.001 |
| Neurologic | 88.0% (85.7%-90.4%) | 80.2% (77.3%-83.1%) | 78.0% (75.0%-81.0%) | <0.001 |
| Neurocognitive | 54.9% (51.3%-58.5%) | 62.3% (58.8%-65.8%) | 70.2% (66.8%-73.5%) | <0.001 |
| Disautonomic | 31.6% (28.3%-35%) | 32.1% (28.7%-35.4%) | 30.1% (26.8%-33.5%) | 0.462 |
| Taste and Smell | 50.3% (46.7%-54%) | 31.5% (28.1%-34.9%) | 14.6% (12.0%-17.1%) | <0.001 |

MEN (n=172)

| Symptoms | Baseline (%) | 22 60d (%) | ≥3 months (%) | P-trend |
| --- | --- | --- | --- | --- |
| Dermatologic | 24.4% (18.0%-30.8%) | 33.7% (26.7%-40.8%) | 28.5% (21.7%-35.2%) | 0.021 |
| Ophtalmologic | 32.0% (25.0%-39.0%) | 34.9% (27.8%-42.0%) | 27.3% (20.7%-34.0%) | 0.096 |
| Urologic_ | 8.1% (4.1%-12.2%) | 5.8% (2.3%-9.3%) | 6.4% (2.7%-10.1%) | 0.538 |
| Sexual | 27.9% (21.2%-34.6%) | 23.8% (17.5%-30.2%) | 22.1% (15.9%-28.3%) | 0.139 |
| Digestive | 51.2% (43.7%-58.6%) | 44.2% (36.8%-51.6%) | 40.7% (33.4%-48.0%) | 0.025 |
| Upper Respiratory Ways | 64.5% (57.4%-71.7%) | 57.6% (50.2%-64.9%) | 50.0% (42.5%-57.5%) | 0.002 |
| Olfactory | 44.2% (36.8%-51.6%) | 31.4% (24.5%-38.3%) | 20.9% (14.9%-27.0%) | <0.001 |
| Ear, Nose, Throat (ENT) | 30.8% (23.9%-37.7%) | 32.6% (25.6%-39.6%) | 29.7% (22.8%-36.5%) | 0.679 |
| Respiratory | 79.1% (73.0%-85.1%) | 72.7% (66.0%-79.3%) | 54.1% (46.6%-61.5%) | <0.001 |
| Cardiac | 54.7% (47.2%-62.1%) | 55.2% (47.8%-62.7%) | 45.9% (38.5%-53.4%) | 0.022 |
| Rheumatologic | 61.1% (53.8%-68.3%) | 59.9% (52.6%-67.2%) | 53.5% (46.0%-60.9%) | 0.141 |
| General | 87.2% (82.2%-92.2%) | 83.1% (77.5%-88.7%) | 72.7% (66.0%-79.3%) | <0.001 |
| Neurological | 78.5% (72.3%-84.6%) | 73.8% (67.3%-80.4%) | 74.4% (67.9%-80.9%) | 0.361 |
| Neurocognitive | 45.4% (37.9%-52.8%) | 55.2% (47.8%-62.7%) | 55.8% (48.4%-63.2%) | 0.009 |
| Disautonomic | 35.5% (28.3%-42.6%) | 31.4% (24.5%-38.3%) | 23.8% (17.5%-30.2%) | 0.005 |
| Taste and Smell | 32.6% (25.6%-39.6%) | 17.4% (11.8%-23.1%) | 6.4% (2.7%-10.1%) | <0.001 |

OTHER (n=6)

| Symptoms | Baseline (%) | 22 60d (%) | ≥3 months (%) | P-trend |
| --- | --- | --- | --- | --- |
| Dermatologic | 33.3% (0%-71%) | 50% (10%-90%) | 50% (10%-90%) | 0.368 |
| Ophtalmologic | 33.3% (0%-71%) | 33.3% (0%-71%) | 33.3% (0%-71%) | 1.000 |
| Urologic_ | 33.3% (0%-71%) | 33.3% (0%-71%) | 16.7% (0%-46.5%) | 0.368 |
| Sexual | 16.7% (0%-46.5%) | 16.7% (0%-46.5%) | 33.3% (0%-71%) | 0.368 |
| Digestive | 50% (10%-90%) | 33.3% (0%-71%) | 50% (10%-90%) | 0.368 |
| Upper Respiratory Ways | 66.7% (29%-100%) | 50% (10%-90%) | 33.3% (0%-71%) | 0.368 |
| Olfactory | 50% (10%-90%) | 33.3% (0%-71%) | 0% (--) | 0.097 |
| Ear, Nose, Throat (ENT) | 16.7% (0%-46.5%) | 16.7% (0%-46.5%) | 16.7% (0%-46.5%) | 1.000 |
| Respiratory | 100% (--) | 50% (10%-90%) | 50% (10%-90%) | 0.105 |
| Cardiac | 50% (10%-90%) | 66.7% (29%-100%) | 66.7% (29%-100%) | 0.368 |
| Rheumatologic | 66.7% (29%-100%) | 66.7% (29%-100%) | 83.3% (53.5%-100%) | 0.368 |
| General | 100% (--) | 83.3% (53.5%-100%) | 83.3% (53.5%-100%) | 0.607 |
| Neurological | 83.3% (53.5%-100%) | 83.3% (53.5%-100%) | 83.3% (53.5%-100%) | 1.000 |
| Neurocognitive | 66.7% (29%-100%) | 50% (10%-90%) | 66.7% (29%-100%) | 0.607 |
| Disautonomic | 50% (10%-90%) | 33.3% (0%-71%) | 33.3% (0%-71%) | 0.607 |
| Taste and Smell | 33.3% (0%-71%) | 16.7% (0%-46.5%) | 0% (--) | 0.223 |
