# Supplementary material for: Characterisation, symptom pattern and symptom clusters from a retrospective cohort of Long COVID patients in primary care in Catalonia
Source: BMC Infect Dis. 2024 Jan 15;24:82. doi: 10.1186/s12879-023-08954-x (PMC10789045; doi:10.1186/s12879-023-08954-x)
Supplement: Supplementary file 6 — Additional file 6: Table S2. Characteristics of end-date of symptoms cohort. [file 12879_2023_8954_MOESM6_ESM.docx]

Table S2. Characteristics of end-date of symptoms cohort.

| **Characteristics** | **Total**  **N (%)** | **Female**  **N(%)** | **Male**  **N(%)** | **p-value** |
| --- | --- | --- | --- | --- |
| **Gender** | 30 (3.1) | 22 (73.3) | 8 (26.7) |  |
| **Age (years)** |  |  |  |  |
| Median (P25-P75) | 42.5 (34.7-51) | 39 (34.5-50.2) | 46.5 (34.2-52.5) |  |
| **Source of income** |  |  |  |  |
| Contract worker | 27 (90) | 20 (90.9) | 7 (87.5) | 0.783 |
| Independent worker with contribution | 2 (6.7) | 1 (4.5) | 1 (12.5) | 0.440 |
| Informal worker (no contract nor contribution) | 0 (0) | 0 (0) | 0 (0) |  |
| Unemployment benefit or other benefits | 0 (0) | 0 (0) | 0 (0) |  |
| Unemployed without benefit nor social benefit | 0 (0) | 0 (0) | 0 (0) |  |
| Household chores or caregiver | 0 (0) | 0 (0) | 0 (0) |  |
| Student | 2 (6.7) | 1 (4.5) | 1 (12.5) | 0.440 |
| Other | 1 (3.3) | 1(4.5) | 0 (0) | 0.540 |
| **Health worker** |  |  |  |  |
| No | 24 (80) | 17 (77.3) | 7 (87.5) | 0.536 |
| Yes | 6 (20) | 5 (22.7) | 1 (12.5) |  |
| **Previous physical activity** |  |  |  | 0.854 |
| Everyday | 2 (6.7) | 1 (4.5) | 1 (12.5) |  |
| 2-3 times a week | 14 (46.7) | 11 (50.0) | 3 (37.5) |  |
| <2-3 times a week | 7 (23.3) | 5 (22.7) | 2 (25.0) |  |
| No practice | 7 (23.3) | 5 (22.7) | 2 (25.0) |  |
| **Smoking habit** |  |  |  |  |
| Smoker | 2 (6,7) | 1 (4,5) | 1 (12.5) | 0.440 |
| Non-smoker | 13 (43.3) | 10 (45.5) | 3 (37.5) | 0.697 |
| Ex-smoker | 7 (23.3) | 4 (18.2) | 3 (37.5) | 0.269 |
| **BMI (kg/m2)** |  |  |  |  |
| Median (P25-P75) | 22.8* (21.5-25.1) | 22.5 (21.5-25.1) | 23.1 (21.1-28.7) |  |
| **Comorbidities** |  |  |  |  |
| No | 16 (53.3) | 12 (54.4) | 4 (50.0) | 0.825 |
| Yes | 14 (46.7) | 10 (45.5) | 4 (50.0) |  |
| **Previous treatments** |  |  |  |  |
| Yes | 8 (26.7) | 6 (27.3) | 2 (25) | 0.901 |
| No | 22 (73.3) | 16 (72.7) | 6 (75) |  |
| **Hospitalization** |  |  |  |  |
| Yes | 3 (10) | 1 (4.5) | 2 (25) | 0.069 |
| **Positive at any time by PCR or TAR*** |  |  |  | 0.064 |
| Never positive | 17 (58.6) | 15 (68.2) | 2 (28.6) |  |
| Sometime positive | 12 (41.4) | 7 (31.8) | 5 (71.4) |  |
| **WAVE** |  |  |  | 0.637 |
| First wave | 19 (86.4) | 15 (88.2) | 4 (80.0) |  |
| Second wave | 3 (13.6) | 2 (11.8) | 1 (20.0) |  |
| Third wave | 0 (0) | 0 (0) | 0 (0) |  |
| Fourth wave | 0 (0) | 0 (0) | 0 (0) |  |
| **Median of days from the onset to the end of symptoms (p25-p75)** | 184 (156.2-389.2) | 183 (151-419.5) | 184 (156-267) |  |

*1 missing
