# Supplementary material for: Characterisation, symptom pattern and symptom clusters from a retrospective cohort of Long COVID patients in primary care in Catalonia
Source: BMC Infect Dis. 2024 Jan 15;24:82. doi: 10.1186/s12879-023-08954-x (PMC10789045; doi:10.1186/s12879-023-08954-x)
Supplement: Supplementary file 7 — Additional file 7: Table S3. Symptoms by sex at baseline. [file 12879_2023_8954_MOESM7_ESM.docx]

**TABLE S3**. Symptoms by sex at baseline.

| **Symptoms at 21 days** | **Women N=727** | **Men**  **N=171** | **Other**  **N=6** | **Total**  **N=904 (1 missing)** | **p-value** |
| --- | --- | --- | --- | --- | --- |
| **Productive cough** | 120 (16.5) | 29 (17.0) | 3 (50.0) | 152 (16.8) | 0.092 |
| **Dry cough** | 396 (54.5) | 79 (46.2) | 2 (33.3) | 477 (52.8) | 0.095 |
| **Shortness of breath** | 439 (60.4) | 100 (58.5) | 3 (50.0) | 542 (60.0) | 0.795 |
| **Shortness of breath at medium efforts** | 357 (49.1) | 77 (45.0) | 3 (50.0) | 437 (48.3) | 0.629 |
| **Shortness of breath at minimum efforts** | 314 (43.2) | 67 (39.2) | 2 (33.3) | 383 (42.4) | 0.573 |
| **Shortness of breath at rest** | 295 (40.6) | 60 (35.1) | 3 (50.0) | 358 (39.6) | 0.365 |
| **Fatigue** | 587 (80.7) | 127 (74.3) | 5 (83.3) | 719 (79.5) | 0.164 |
| **General malaise** | 590 (81.2) | 124 (72.5) | 5 (83.3) | 719 (79.5) | **0.041** |
| **Dysthermia** | 498 (68.5) | 117 (68.4) | 4 (66.7) | 619 (68.5) | 0.995 |
| **Temperature <37** | 103 (14.2) | 23 (13.5) | 1 (16.7) | 127 (14.0) | 0.954 |
| **Temperature**  **37-37,5** | 238 (32.7) | 42 (24.6) | 2 (33.3) | 282 (31.2) | 0.115 |
| **Temperature 37,6-38** | 206 (28.3) | 48 (28.1) | 1 (16.7) | 255 (28.2) | 0.818 |
| **Temperature major 38** | 166 (22.8) | 46 (26.9) | 1 (16.7) | 213 (23.6) | 0.489 |
| **Diarrhoea** | 320 (44.0) | 65 (38.0) | 4 (66.7) | 389 (43.0) | 0.182 |
| **Palpitations** | 322 (44.3) | 57 (33.3) | 3 (50.0) | 382 (42.3) | **0.031** |
| **Chest pain** | 312 (42.9) | 63 (36.8) | 2 (33.3) | 377 (41.7) | 0.321 |
| **Oppressive chest pain** | 235 (32.3) | 49 (28.7) | 2 (33.3) | 286 (31.6) | 0.647 |
| **Burning chest pain** | 117 (16.1) | 19 (11.1) | 1 (16.7) | 137 (15.2) | 0.261 |
| **Other kind of chest pain** | 70 (9.6) | 15 (8.8) | 0 (0) | 85 (9.4) | 0.689 |
| **Muscle pain** | 497 (68.4) | 98 (57.3) | 3 (50.0) | 598 (66.2) | **0.016** |
| **Joint pain** | 404 (55.6) | 77 (45.0) | 3 (50.0) | 484 (53.5) | **0.045** |
| **Back pain** | 399 (54.9) | 65 (38.0) | 3 (50.0) | 467 (51.7) | **0.000** |
| **Headache** | 549 (75.5) | 100 (58.5) | 5 (83.3) | 654 (72.3) | **0.000** |
| **Dizziness** | 294 (40.4) | 57 (33.3) | 2 (33.3) | 353 (39.0) | 0.221 |
| **Tingling** | 235 (32.3) | 43 (25.1) | 1 (16.7) | 279 (30.9) | 0.141 |
| **Lack of Concentration** | 362 (49.8) | 68 (39.8) | 4 (66.7) | 434 (48.0) | **0.040** |
| **Oversights** | 207 (28.5) | 39 (22.8) | 2 (33.3) | 248 (27.4) | 0.311 |
| **Difficulties in remembering things** | 123 (16.9) | 18 (10.5) | 1 (16.7) | 142 (15.7) | 0.118 |
| **Aphonia** | 122 (16.8) | 35 (20.5) | 0 (0) | 157 (17.4) | 0.275 |
| **Dysphonia** | 123 (16.9) | 36 (21.1) | 1 (16.7) | 160 (17.7) | 0.443 |
| **Scrape in your throat** | 185 (25.4) | 45 (26.3) | 0 (0) | 230 (25.4) | 0.347 |
| **Nasal congestion** | 222 (30.5) | 40 (23.4) | 3 (50.0) | 265 (29.3) | 0.097 |
| **Nose bleeding** | 18 (2.5) | 10 (5.8) | 1 (16.7) | 29 (3.2) | **0.014** |
| **Mucus in the nose** | 108 (14.9) | 19 (11.1) | 2 (33.3) | 129 (14.2) | 0.184 |
| **Itchy nose** | 95 (13.1) | 23 (13.5) | 1 (16.7) | 119 (13.2) | 0.960 |
| **Dry nose** | 145 (19.9) | 33 (19.3) | 1 (16.7) | 179 (19.8) | 0.964 |
| **Sneezing** | 131 (18.0) | 23 (13.5) | 2 (33.3) | 156 (17.3) | 0.210 |
| **Ear ache** | 94 (12.9) | 14 (8.2) | 1 (16.7) | 109 (12.1) | 0.217 |
| **Tinnitus** | 85 (11.7) | 16 (9.4) | 1 (16.7) | 102 (11.3) | 0.628 |
| **Sore throat** | 283 (38.9) | 50 (29.2) | 3 (50.0) | 336 (37.2) | **0.050** |
| **Itchy throat** | 157 (21.6) | 30 (17.5) | 2 (33.3) | 189 (20.9) | 0.379 |
| **Blood taste on your throat** | 73 (10.0) | 14 (8.2) | 1 (16.7) | 88 (9.7) | 0.647 |
| **Dry throat** | 201 (27.6) | 49 (28.7) | 1 (16.7) | 251 (27.8) | 0.802 |
| **Sputum** | 52 (7.2) | 15 (8.8) | 1 (16.7) | 68 (7.5) | 0.536 |
| **Blue lips** | 21 (2.9) | 2 (1.2) | 0 (0) | 23 (2.5) | 0.405 |
| **Incomplete inspiration** | 308 (42.4) | 70 (40.9) | 3 (50.0) | 381 (42.1) | 0.874 |
| **Abnormal breathing** | 162 (22.3) | 39 (22.8) | 2 (33.3) | 203 (22.5) | 0.806 |
| **Low oxygen saturation <95%** | 157 (21.6) | 37 (21.6) | 2 (33.3) | 196 (21.7) | 0.785 |
| **Inappetence** | 308 (42.4) | 53 (31.0) | 1 (16.7) | 362 (40.0) | **0.012** |
| **Weight loss** | 311 (42.8) | 67 (39.2) | 1 (16.7) | 379 (41.9) | 0.314 |
| **Muscle weakness** | 435 (59.8) | 90 (52.6) | 2 (33.3) | 527 (58.3) | 0.105 |
| **Shivers** | 294 (40.4) | 57 (33.3) | 2 (33.3) | 353 (39.0) | 0.221 |
| **Inappropriate perspiration** | 169 (23.2) | 46 (26.9) | 2 (33.3) | 217 (24.0) | 0.522 |
| **Abdominal pain** | 198 (27.2) | 34 (19.9) | 2 (33.3) | 234 (25.9) | 0.130 |
| **Stomach ache** | 175 (24.1) | 31 (18.1) | 3 (50.0) | 209 (23.1) | 0.074 |
| **Nausea** | 231 (31.8) | 36 (21.1) | 1 (16.7) | 268 (29.6) | **0.017** |
| **Vomiting** | 114 (15.7) | 16 (9.4) | 0 (0) | 130 (14.4) | 0.064 |
| **Mucus in the stool** | 48 (6.6) | 6 (3.5) | 1 (16.7) | 55 (6.1) | 0.174 |
| **Blood in the stool** | 11 (1.5) | 4 (2.3) | 0 (0) | 15 (1.7) | 0.711 |
| **Liquid stool** | 217 (29.8) | 48 (28.1) | 2 (33.3) | 267 (29.5) | 0.881 |
| **Intestinal sounds** | 131 (18.0) | 24 (14.0) | 2 (33.3) | 157 (17.4) | 0.272 |
| **Flatulence** | 157 (21.6) | 38 (22.2) | 2 (33.3) | 197 (21.8) | 0.777 |
| **Oral aphthae** | 117 (16.1) | 17 (9.9) | 0(0) | 134 (14.8) | 0.074 |
| **Oral herpes** | 58 (8.0) | 12 (7.0) | 0 (0) | 70 (7.7) | 0.710 |
| **Dry eyes** | 138 (19.0) | 22 (12.9) | 2 (33.3) | 162 (17.9) | 0.106 |
| **Painful eyes** | 143 (19.7) | 22 (12.9) | 1 (16.7) | 166 (18.4) | 0.117 |
| **Conjunctivitis** | 74 (10.2) | 10 (5.8) | 1 (16.7) | 85 (9.4) | 0.181 |
| **Red eyes** | 69 (9.5) | 17 (9.9) | 0 (0) | 86 (9.5) | 0.716 |
| **Blurred vision** | 115 (15.8) | 24 (14.0) | 1 (16.7) | 140 (15.5) | 0.842 |
| **Diplopia** | 26 (3.6) | 4 (2.3) | 0(0) | 30 (3.3) | 0.648 |
| **Photophobia** | 115 (15.8) | 18 (10.5) | 1 (16.7) | 134 (14.8) | 0.214 |
| **High Blood Pressure** | 68 (9.4) | 14 (8.2) | 1 (16.7) | 83 (9.2) | 0.729 |
| **Orthostatic hypotension** | 108 (14.9) | 11 (6.4) | 1 (16.7) | 120 (13.3) | **0.014** |
| **Tachycardia** | 236 (32.5) | 44 (25.7) | 3 (50.0) | 283 (31.3) | 0.142 |
| **Bradycardia** | 21 (2.9) | 6 (3.5) | 0 (0) | 27 (3.0) | 0.831 |
| **Arthritis (joint inflammation)** | 43 (5.9) | 3 (1.8) | 1 (16.7) | 47 (5.2) | **0.039** |
| **Neck pain** | 129 (17.7) | 26 (15.2) | 1 (16.7) | 156 (17.3) | 0.731 |
| **Right hypochondrium pain** | 95 (13.1) | 14 (8.2) | 2 (33.3) | 111 (12.3) | 0.062 |
| **Left hypochondrium pain** | 74 (10.2) | 18 (10.5) | 1 (16.7) | 93 (10.3) | 0.867 |
| **Pain from old injuries** | 53 (7.3) | 13 (7.6) | 0 (0) | 66 (7.3) | 0.781 |
| **Pins and needles pain** | 158 (21.7) | 26 (15.2) | 1 (16.7) | 185 (20.5) | 0.159 |
| **Rib pain** | 116 (16.0) | 19 (11.1) | 1 (16.7) | 136 (15.0) | 0.279 |
| **Ageusia** | 388 (53.4) | 61 (35.7) | 3 (50.0) | 452 (50.0) | **0.000** |
| **Anosmia** | 402 (55.3) | 68 (39.8) | 2 (33.3) | 472 (52.2) | **0.001** |
| **Cacosmia** | 103 (14.2) | 16 (9.4) | 1 (16.7) | 120 (13.3) | 0.241 |
| **Phantosmia** | 67 (9.2) | 10 (5.8) | 0 (0) | 77 (8.5) | 0.276 |
| **Loss of hearing** | 54 (7.4) | 8 (4.7) | 0(0) | 62 (6.9) | 0.353 |
| **Excessive hearing** | 54 (7.4) | 12 (7.0) | 0(0) | 66 (7.3) | 0.775 |
| **Hypoesthesia** | 47 (6.5) | 9 (5.3) | 0(0) | 56 (6.2) | 0.690 |
| **Cramps** | 106 (14.6) | 23 (13.5) | 2 (33.3) | 131 (14.5) | 0.392 |
| **Fasciculations** | 71 (9.8) | 12 (7.0) | 0 (0) | 83 (9.2) | 0.394 |
| **Incoordination** | 52 (7.2) | 12 (7.0) | 0 (0) | 64 (7.1) | 0.793 |
| **Difficulty in fine motor skills** | 63 (8.7) | 11 (6.4) | 0 (0) | 74 (8.2) | 0.483 |
| **Disorientation** | 80 (11.0) | 9 (5.3) | 1 (16.7) | 90 (10.0) | 0.067 |
| **Anomia** | 145 (19.9) | 23 (12.5) | 1 (16.7) | 169 (18.7) | 0.145 |
| **Alexia** | 82 (11.3) | 14 (8.2) | 0 (0) | 96 (10.6) | 0.348 |
| **Trembling** | 98 (13.5) | 25 (14.6) | 3 (50.0) | 126 (13.9) | **0.035** |
| **Convulsions** | 15 (2.1) | 3 (1.8) | 0 (0) | 18 (2.0) | 0.909 |
| **Onset insomnia** | 214 (29.4) | 40 (23.4) | 4 (66.7) | 258 (28.5) | **0.034** |
| **Maintenance insomnia** | 188 (25.9) | 39 (22.8) | 4 (66.7) | 231 (25.6) | **0.049** |
| **Hair loss** | 203 (27.9) | 17 (9.9) | 1 (16.7) | 221 (24.4) | **0.000** |
| **Increased body odour** | 63 (8.7) | 13 (7.6) | 0 (0) | 76 (8.4) | 0.685 |
| **Dry skin** | 169 (23.2) | 18 (10.5) | 2 (33.3) | 189 (20.9) | **0.001** |
| **Itchy skin** | 128 (17.6) | 23 (13.5) | 1 (16.7) | 152 (16.8) | 0.425 |
| **Rash on the skin** | 133 (18.3) | 21 (12.3) | 2 (33.3) | 156 (17.3) | 0.100 |
| **Erythema pernio** | 20 (2.8) | 1 (0.6) | 0 (0) | 21 (2.3) | 0.222 |
| **Livedo reticularis** | 11 (1.5) | 1 (0.6) | 0 (0) | 12 (1.3) | 0.609 |
| **Menstrual alterations** | 91 (12.5) | 1 (0.6) | 0 (0) | 92 (10.2) | **0.000** |
| **Changes in the length of the cycle** | 59 (8.1) | 1 (0.6) | 0 (0) | 60 (6.6) | **0.001** |
| **Changes in the volume of the cycle** | 47 (6.5) | 1 (0.6) | 0 (0) | 48 (5.3) | **0.007** |
| **Dysmenorrhea** | 64 (8.8) | 1 (0.6) | 0 (0) | 65 (7.2) | **0.001** |
| **Vaginal discomfort** | 46 (6.3) | 0 (0) | (0) | 46 (5.1) | **0.003** |
| **Genital discomfort** | 1 (0.1) | 6 (3.5) | 0 (0) | 7 (0.8) | **0.000** |
| **Dysuria** | 54 (7.4) | 11 (6.4) | 2 (33.3) | 67 (7.4) | **0.047** |
| **Frequent micturition** | 87 (12.0) | 22 (12.9) | 2 (33.3) | 111 (12.3) | 0.274 |
| **Low sexual desire** | 187 (25.7) | 47 (27.5) | 1 (16.7) | 235 (26.0) | 0.780 |
| **Sexual Dysfunction** | 44 (6.1) | 5 (2.9) | 1 (16.7) | 50 (5.5) | 0.134 |
