# Supplementary material for: Characterisation, symptom pattern and symptom clusters from a retrospective cohort of Long COVID patients in primary care in Catalonia
Source: BMC Infect Dis. 2024 Jan 15;24:82. doi: 10.1186/s12879-023-08954-x (PMC10789045; doi:10.1186/s12879-023-08954-x)
Supplement: Supplementary file 8 — Additional file 8: Table S4. Symptoms by sex at 22-60 days. [file 12879_2023_8954_MOESM8_ESM.docx]

**TABLE S4.** Symptoms by sex at 22-60 days.

| **Symptoms at 22-60 days** | **Women N=727** | **Men**  **N=171** | **Other**  **N=6** | **Total**  **N=904** | **p-value** |
| --- | --- | --- | --- | --- | --- |
| **Productive cough** | 71 (9.8) | 13 (7.6) | 0 (0) | 84 (9.3) | 0.500 |
| **Dry cough** | 200 (27.5) | 42 (24.6) | 0 (0) | 242 (26.8) | 0.244 |
| **Shortness of breath** | 347 (47.7) | 84 (49.1) | 1 (16.7) | 432 (47.8) | 0.293 |
| **Shortness of breath at medium efforts** | 361 (49.7) | 82 (48.0) | 2 (33.3) | 445 (49.2) | 0.680 |
| **Shortness of breath at minimum efforts** | 275 (37.8) | 57 (33.3) | 1 (16.7) | 333 (36.8) | 0.323 |
| **Shortness of breath at rest** | 183 (25.2) | 45 (26.3) | 1 (16.7) | 229 (25.3) | 0.846 |
| **Fatigue** | 540 (74.3) | 113 (66.1) | 4 (66.7) | 657 (72.7) | 0.091 |
| **General malaise** | 402 (55.3) | 85 (49.7) | 3 (50.0) | 490 (54.2) | 0.410 |
| **Dysthermia** | 255 (35.1) | 41 (24.0) | 1 (16.7) | 297 (32.9) | **0.015** |
| **Temperature <37** | 112 (15.4) | 23 (13.5) | 1 (16.7) | 136 (15.0) | 0.808 |
| **Temperature**  **37-37,5** | 183 (25.2) | 26 (15.2) | 0 (0) | 209 (23.1) | **0.008** |
| **Temperature 37,6-38** | 55 (7.6) | 8 (4.7) | 1 (16.7) | 64 (7.1) | 0.273 |
| **Temperature major 38** | 19 (2.6) | 2 (1.2) | 1 (16.7) | 22 (2.4) | **0.041** |
| **Diarrhoea** | 166 (22.8) | 39 (22.8) | 2 (33.3) | 207 (22.9) | 0.830 |
| **Palpitations** | 315 (43.3) | 55 (32.2) | 4 (66.7) | 374 (41.4) | **0.013** |
| **Chest pain** | 267 (36.7) | 51 (29.8) | 1 (16.7) | 319 (35.3) | 0.149 |
| **Oppressive chest pain** | 203 (27.9) | 171 (26.3) | 1 (16.7) | 249 (27.5) | 0.764 |
| **Burning chest pain** | 95 (13.1) | 19 (11.1) | 1 (16.7) | 115 (12.7) | 0.755 |
| **Other kind of chest pain** | 65 (8.9) | 13 (7.6) | 0 (0) | 78 (8.6) | 0.642 |
| **Muscle pain** | 401 (55.2) | 83 (48.5) | 2 (33.3) | 486 (53.8) | 0.178 |
| **Joint pain** | 337 (46.4) | 68 (39.8) | 2 (33.3) | 407 (45.0) | 0.251 |
| **Back pain** | 353 (48.6) | 63 (36.8) | 3 (50.0) | 419 (46.3) | **0.022** |
| **Headache** | 399 (54.9) | 72 (42.1) | 5 (83.3) | 476 (52.7) | **0.003** |
| **Dizziness** | 239 (32.9) | 52 (30.4) | 1 (16.7) | 292 (32.3) | 0.589 |
| **Tingling** | 245 (33.7) | 45 (26.3) | 1 (16.7) | 291 (32.2) | 0.127 |
| **Lack of Concentration** | 405 (55.7) | 75 (43.9) | 3 (50.0) | 483 (53.4) | **0.020** |
| **Oversights** | 321 (44.2) | 61 (35.7) | 2 (33.3) | 384 (42.5) | 0.118 |
| **Difficulties in remembering things** | 186 (25.6) | 30 (17.5) | 1 (16.7) | 217 (24.0) | 0.079 |
| **Aphonia** | 101 (13.9) | 23 (13.5) | 0 (0) | 124 (13.7) | 0.612 |
| **Dysphonia** | 112 (15.4) | 29 (17.0) | 1 (16.7) | 142 (15.7) | 0.880 |
| **Scrape in your throat** | 153 (21.0) | 35 (20.5) | 0 (0) | 188 (20.8) | 0.446 |
| **Nasal congestion** | 125 (17.2) | 29 (17.0) | 1 (16.7) | 155 (17.1) | 0.997 |
| **Nose bleeding** | 20 (2.8) | 6 (3.5) | 1 (16.7) | 27 (3.0) | 0.124 |
| **Mucus in the nose** | 64 (8.8) | 9 (5.3) | 1 (16.7) | 74 (8.2) | 0.236 |
| **Itchy nose** | 66 (9.1) | 14 (8.2) | 1 (16.7) | 81 (9.0) | 0.750 |
| **Dry nose** | 124 (17.1) | 29 (17.0) | 1 (16.7) | 154 (17.0) | 0.999 |
| **Sneezing** | 84 (11.6) | 20 (11.7) | 1 (16.7) | 105 (11.6) | 0.926 |
| **Earache** | 108 (14.9) | 23 (13.5) | 1 (16.7) | 132 (14.6) | 0.887 |
| **Tinnitus** | 118 (16.2) | 28 (16.4) | 1 (16.7) | 147 (16.3) | 0.999 |
| **Sore throat** | 166 (22.8) | 29 (17.0) | 1 (16.7) | 196 (21.7) | 0.234 |
| **Itchy throat** | 113 (15.5) | 27 (15.8) | 2 (33.3) | 142 (15.7) | 0.491 |
| **Blood taste on your throat** | 56 (7.7) | 8 (4.7) | 1 (16.7) | 65 (7.2) | 0.258 |
| **Dry throat** | 181 (24.9) | 44 (25.7) | 1 (16.7) | 226 (25.0) | 0.871 |
| **Sputum** | 45 (6.2) | 12 (7.0) | 1 (16.7) | 58 (6.4) | 0.545 |
| **Blue lips** | 15 (2.1) | 1 (0.6) | 0 (0) | 16 (1.8) | 0.397 |
| **Incomplete inspiration** | 265 (36.5) | 58 (33.9) | 3 (50.0) | 326 (36.1) | 0.639 |
| **Abnormal breathing** | 144 (19.8) | 31 (18.1) | 2 (33.3) | 177 (19.6) | 0.615 |
| **Low oxygen saturation <95%** | 82 (11.3) | 15 (8.8) | 0 (0) | 97 (10.7) | 0.442 |
| **Inappetence** | 184 (25.3) | 27 (15.8) | 1 (16.7) | 212 (23.5) | **0.028** |
| **Weight loss** | 178 (24.5) | 48 (28.1) | 1 (16.7) | 227 (25.1) | 0.556 |
| **Muscle weakness** | 379 (52.1) | 76 (44.4) | 2 (33.3) | 457 (50.6) | 0.136 |
| **Shivers** | 156 (21.5) | 23 (13.5) | 1 (16.7) | 180 (19.9) | 0.061 |
| **Inappropriate perspiration** | 152 (20.9) | 27 (15.8) | 1 (16.7) | 180 (19.9) | 0.314 |
| **Abdominal pain** | 162 (22.3) | 29 (17.0) | 2 (33.3) | 193 (21.3) | 0.240 |
| **Stomach ache** | 147 (20.2) | 27 (15.8) | 2 (33.3) | 176 (19.5) | 0.290 |
| **Nausea** | 151 (20.8) | 21 (12.3) | 1 (16.7) | 173 (19.1) | **0.039** |
| **Vomiting** | 43 (5.9) | 8 (4.7) | 0 (0) | 51 (5.6) | 0.684 |
| **Mucus in the stool** | 45 (6.2) | 4 (2.3) | 1 (16.7) | 50 (5.5) | 0.068 |
| **Blood in the stool** | 11 (1.5) | 7 (4.1) | 0 (0) | 18 (2.0) | 0.089 |
| **Liquid stool** | 135 (18.6) | 28 (16.4) | 2 (33.3) | 165 (18.3) | 0.505 |
| **Intestinal sounds** | 133 (18.3) | 20 (11.7) | 2 (33.3) | 155 (17.1) | 0.069 |
| **Flatulence** | 177 (24.3) | 45 (26.3) | 2 (33.3) | 224 (24.8) | 0.769 |
| **Oral aphthae** | 112 (15.4) | 11 (6.4) | 0 (0) | 123 (13.6) | **0.005** |
| **Oral herpes** | 71 (9.8) | 10 (5.8) | 0 (0) | 81 (9.0) | 0.202 |
| **Dry eyes** | 144 (19.8) | 17 (9.9) | 2 (33.3) | 163 (18.0) | **0.006** |
| **Painful eyes** | 150 (20.6) | 25 (14.6) | 1 (16.7) | 176 (19.5) | 0.200 |
| **Conjunctivitis** | 61 (8.4) | 10 (5.8) | 1 (16.7) | 72 (8.0) | 0.398 |
| **Red eyes** | 62 (8.5) | 14 (8.2) | 0 (0) | 76 (8.4) | 0.750 |
| **Blurred vision** | 127 (17.5) | 31 (18.1) | 0 (0) | 158 (17.5) | 0.517 |
| **Diplopia** | 26 (3.6) | 3 (1.8) | 0 (0) | 29 (3.2) | 0.432 |
| **Photophobia** | 108 (14.9) | 17 (9.9) | 1 (16.7) | 126 (13.9) | 0.244 |
| **High Blood Pressure** | 68 (9.4) | 20 (11.7) | 2 (33.3) | 90 (10.0) | 0.104 |
| **Orthostatic hypotension** | 109 (15.0) | 11 (6.4) | 1 (16.7) | 121 (13.4) | **0.012** |
| **Tachycardia** | 251 (34.5) | 47 (27.5) | 3 (50.0) | 301 (33.3) | 0.146 |
| **Bradycardia** | 31 (4.3) | 8 (4.7) | 0 (0) | 39 (4.3) | 0.848 |
| **Arthritis (joint inflammation)** | 48 (6.6) | 3 (1.8) | 1 (16.7) | 52 (5.8) | **0.026** |
| **Neck pain** | 142 (19.5) | 33 (19.3) | 1 (16.7) | 176 (19.5) | 0.983 |
| **Right hypochondrium pain** | 107 (14.7) | 17 (9.9) | 2 (33.3) | 126 (13.9) | 0.104 |
| **Left hypochondrium pain** | 80 (11.0) | 17 (9.9) | 1 (16.7) | 98 (10.8) | 0.830 |
| **Pain from old injuries** | 63 (8.7) | 14 (8.2) | 0 (0) | 77 (8.5) | 0.740 |
| **Pins and needles pain** | 150 (20.6) | 20 (11.7) | 1 (16.7) | 171 (18.9) | **0.027** |
| **Rib pain** | 115 (15.8) | 20 (11.7) | 1 (16.7) | 136 (15.0) | 0.396 |
| **Ageusia** | 247 (34.0) | 34 (19.9) | 2 (33.3) | 283 (31.3) | **0.002** |
| **Anosmia** | 278 (38.2) | 45 (26.3) | 1 (16.7) | 324 (35.8) | **0.009** |
| **Cacosmia** | 138 (19.0) | 16 (9.4) | 1 (16.7) | 155 (17.1) | **0.011** |
| **Phantosmia** | 97 (13.3) | 13 (7.6) | 0 (0) | 110 (12.2) | 0.078 |
| **Loss of hearing** | 72 (9.9) | 12 (7.0) | 0 (0) | 84 (9.3) | 0.370 |
| **Excessive hearing** | 69 (9.5) | 12 (7.0) | 0 (0) | 81 (9.0) | 0.442 |
| **Hypoesthesia** | 61 (8.4) | 11 (6.4) | 0 (0) | 72 (8.0) | 0.536 |
| **Cramps** | 140 (19.3) | 26 (15.2) | 2 (33.3) | 168 (18.6) | 0.306 |
| **Fasciculations** | 96 (13.2) | 17 (9.9) | 0 (0) | 113 (12.5) | 0.331 |
| **Incoordination** | 76 (10.5) | 12 (7.0) | 0 (0) | 88 (9.7) | 0.285 |
| **Difficulty in fine motor skills** | 80 (11.0) | 13 (7.6) | 0 (0) | 93 (10.3) | 0.297 |
| **Disorientation** | 96 (13.2) | 12 (7.0) | 1 (16.7) | 109 (12.1) | 0.077 |
| **Anomia** | 230 (31.6) | 35 (20.5) | 1 (16.7) | 266 (29.4) | **0.012** |
| **Alexia** | 103 (14.2) | 17 (9.9) | 0 (0) | 120 (13.3) | 0.215 |
| **Trembling** | 77 (10.6) | 17 (9.9) | 2 (33.3) | 96 (10.6) | 0.188 |
| **Convulsions** | 12 (1.7) | 4 (2.3) | 0 (0) | 16 (1.6) | 0.784 |
| **Onset insomnia** | 254 (34.9) | 38 (22.2) | 2 (33.3) | 294 (32.5) | **0.006** |
| **Maintenance insomnia** | 253 (34.8) | 49 (28.7) | 3 (50.0) | 305 (33.7) | 0.217 |
| **Hair loss** | 296 (40.7) | 24 (14.0) | 2 (33.3) | 322 (35.6) | **0.000** |
| **Increased body odour** | 76 (10.5) | 16 (9.4) | 0 (0) | 92 (10.2) | 0.648 |
| **Dry skin** | 200 (27.5) | 22 (12.9) | 2 (33.3) | 224 (24.8) | **0.000** |
| **Itchy skin** | 148 (20.4) | 29 (17.0) | 1 (16.7) | 178 (19.7) | 0.593 |
| **Rash on the skin** | 134 (18.4) | 24 (14.0) | 1 (16.7) | 159 (17.6) | 0.397 |
| **Erythema pernio** | 16 (2.2) | 4 (2.3) | 0 (0) | 20 (2.2) | 0.928 |
| **Livedo reticularis** | 13 (1.8) | 1 (0.6) | 0 (0) | 14 (1.5) | 0.494 |
| **Menstrual alterations** | 110 (15.1) | 1 (0.6) | 0 (0) | 111 (12.3) | **0.000** |
| **Changes in the length of the cycle** | 78 (10.7) | 1 (0.6) | 0 (0) | 79 (8.7) | **0.000** |
| **Changes in the volume of the cycle** | 65 (8.9) | 1 (0.6) | 0 (0) | 66 (7.3) | **0.001** |
| **Dysmenorrhea** | 92 (12.7) | 1 (0.6) | 0 (0) | 93 (10.3) | **0.000** |
| **Vaginal discomfort** | 71 (9.8) | 0 (0) | 0 (0) | 71 (7.9) | **0.000** |
| **Genital discomfort** | 2 (0.3) | 7 (4.1) | 0 (0) | 9 (1.0) | **0.000** |
| **Dysuria** | 57 (7.8) | 7 (4.1) | 2 (33.3) | 66 (7.3) | **0.012** |
| **Frequent micturition** | 111 (15.3) | 25 (14.6) | 2 (33.3) | 138 (15.3) | 0.456 |
| **Low sexual desire** | 188 (25.9) | 40 (23.4) | 1 (16.7) | 229 (25.3) | 0.710 |
| **Sexual Disfunction** | 54 (7.4) | 8 (4.7) | 1 (16.7) | 63 (7.0) | 0.288 |
