# Supplementary material for: Characterisation, symptom pattern and symptom clusters from a retrospective cohort of Long COVID patients in primary care in Catalonia
Source: BMC Infect Dis. 2024 Jan 15;24:82. doi: 10.1186/s12879-023-08954-x (PMC10789045; doi:10.1186/s12879-023-08954-x)
Supplement: Supplementary file 9 — Additional file 9: Table S5. Symptoms by sex at ≥ 3 months. [file 12879_2023_8954_MOESM9_ESM.docx]

**TABLE S5**. Symptoms by sex at ≥ 3 months.

| **Symptoms ≥3 months** | **Women**  **N=727** | **Men**  **N=171** | **Other**  **N=6** | **Total**  **N=904** | **p-value** |
| --- | --- | --- | --- | --- | --- |
| **Productive cough** | 37 (5.1) | 5 (2.9) | 0 (0) | 42 (4.6) | 0.415 |
| **Dry cough** | 88 (12.1) | 23 (13.5) | 0 (0) | 111 (12.3) | 0.583 |
| **Shortness of breath** | 220 (30.3) | 50 (29.2) | 2 (33.3) | 272 (30.1) | 0.952 |
| **Shortness of breath at medium efforts** | 344 (47.3) | 68 (39.8) | 2 (33.3) | 414 (45.8) | 0.169 |
| **Shortness of breath at minimum efforts** | 174 (23.9) | 32 (18.7) | 2 (33.3) | 208 (23.0) | 0.287 |
| **Shortness of breath at rest** | 88 (12.1) | 23 (13.5) | 0 (0) | 111 (12.3) | 0.583 |
| **Fatigue** | 503 (69.2) | 102 (59.6) | 5 (83.3) | 610 (67.5) | **0.040** |
| **General malaise** | 283 (38.9) | 58 (33.9) | 2 (33.3) | 343 (37.9) | 0.465 |
| **Dysthermia** | 147 (20.2) | 17 (9.9) | 0 (0) | 164 (18.1) | **0.004** |
| **Temperature <37** | 175 (24.1) | 36 (21.1) | 1 (16.7) | 212 (23.5) | 0.651 |
| **Temperature**  **37-37,5** | 73 (10.0) | 6 (3.5) | 1 (16.7) | 80 (8.8) | **0.020** |
| **Temperature 37,6-38** | 10 (1.4) | 0 (0) | 0(0) | 10 (1.1) | 0.292 |
| **Temperature major 38** | 2 (0.3) | 0 (0) | 0 (0) | 2 (‘.2) | 0.783 |
| **Diarrhoea** | 74 (10.2) | 18 (10.5) | 1 (16.7) | 93 (10.3) | 0.867 |
| **Palpitations** | 252 (34.7) | 32 (18.7) | 3 (50.0) | 287 (31.7) | **0.000** |
| **Chest pain** | 178 (24.5) | 33 (19.3) | 2 (33.3) | 213 (23.6) | 0.303 |
| **Oppressive chest pain** | 140 (19.3) | 21 (12.3) | 1 (16.7) | 162 (17.9) | 0.101 |
| **Burning chest pain** | 58 (8.0) | 10 (5.8) | 2 (33.3) | 70 (7.7) | **0.040** |
| **Other kind of chest pain** | 57 (7.8) | 19 (11.1) | 0 (0) | 76 (8.4) | 0.290 |
| **Muscle pain** | 339 (46.6) | 74 (43.3) | 2 (33.3) | 415 (45.9) | 0.603 |
| **Joint pain** | 315 (43.3) | 60 (35.1) | 3 (50.0) | 378 (41.8) | 0.133 |
| **Back pain** | 360 (49.5) | 63 (36.8) | 4 (66.7) | 427 (47.2) | **0.007** |
| **Headache** | 340 (46.8) | 53 (31.0) | 4 (66.7) | 397 (43.9) | **0.000** |
| **Dizziness** | 216 (29.7) | 32 (18.7) | 1 (16.7) | 249 (27.5) | **0.013** |
| **Tingling** | 239 (32.9) | 43 (25.1) | 1 (16.7) | 283 (31.3) | 0.108 |
| **Lack of Concentration** | 424 (58.3) | 71 (41.5) | 4 (66.7) | 499 (55.2) | **0.000** |
| **Oversights** | 422 (58.0) | 71 (41.5) | 3 (50.0) | 496 (54.9) | **0.000** |
| **Difficulties in remembering things** | 235 (32.3) | 32 (18.7) | 1 (16.7) | 268 (29.6) | **0.002** |
| **Aphonia** | 55 (7.6) | 11 (6.4) | 0 (0) | 66 (7.3) | 0.691 |
| **Dysphonia** | 83 (11.4) | 20 (11.7) | 0 (0) | 103 (11.4) | 0.675 |
| **Scrape in your throat** | 125 (17.2) | 28 (16.4) | 0 (0) | 153 (16.9) | 0.523 |
| **Nasal congestion** | 114 (15.7) | 26 (15.2) | 0 (0) | 140 (15.5) | 0.568 |
| **Nose bleeding** | 16 (2.2) | 4 (2.3) | 0 (0) | 20 (2.2) | 0.928 |
| **Mucus in the nose** | 62 (8.5) | 11 (6.4) | 1 (16.7) | 74 (8.2) | 0.500 |
| **Itchy nose** | 65 (8.9) | 13 (7.6) | 0 (0) | 78 (8.6) | 0.642 |
| **Dry nose** | 129 (17.7) | 24 (14.0) | 1 (16.7) | 154 (17.0) | 0.510 |
| **Sneezing** | 93 (12.8) | 24 (14.0) | 1 (16.7) | 118 (13.1) | 0.879 |
| **Earache** | 75 (10.3) | 9 (5.3) | 0(0) | 84 (9.3) | 0.090 |
| **Tinnitus** | 123 (16.9) | 33 (19.3) | 1 (16.7) | 157 (17.4) | 0.760 |
| **Sore throat** | 115 (15.8) | 18 (10.5) | 1 (16.7) | 134 (14.8) | 0.214 |
| **Itchy throat** | 81 (11.1) | 18 (10.5) | 1 (16.7) | 100 (11.1) | 0.884 |
| **Blood taste on your throat** | 42 (5.8) | 4 (2.3) | 0 (0) | 46 (5.1) | 0.156 |
| **Dry throat** | 160 (22.0) | 37 (21.6) | 2 (33.3) | 199 (22.0) | 0.794 |
| **Sputum** | 28 (3.9) | 8 (4.7) | 0 (0) | 36 (4.0) | 0.780 |
| **Blue lips** | 10 (1.4) | 0 (0) | 0 (0) | 10 (1.1) | 0.292 |
| **Incomplete inspiration** | 200 (27.5) | 38 (22.2) | 1 (16.7) | 239 (26.4) | 0.319 |
| **Abnormal breathing** | 105 (14.4) | 18 (10.5) | 1 (16.7) | 124 (13.7) | 0.399 |
| **Low oxygen saturation <95%** | 38 (5.2) | 9 (5.3) | 1 (16.7) | 48 (5.3) | 0.461 |
| **Inappetence** | 99 (13.6) | 11 (6.4) | 1 (16.7) | 111 (12.3) | **0.034** |
| **Weight loss** | 81 (11.1) | 14 (8.2) | 0 (0) | 95 (10.5) | 0.369 |
| **Muscle weakness** | 312 (42.9) | 62 (36.3) | 2 (33.3) | 376 (41.6) | 0.260 |
| **Shivers** | 123 (16.9) | 17 (9.9) | 1 (16.7) | 141 (15.6) | 0.077 |
| **Inappropriate perspiration** | 108 (14.9) | 15 (8.8) | 1 (16.7) | 124 (13.7) | 0.112 |
| **Abdominal pain** | 145 (19.9) | 19 (11.1) | 2 (33.3) | 166 (18.4) | **0.017** |
| **Stomach ache** | 130 (17.9) | 23 (13.5) | 2 (33.3) | 155 (17.1) | 0.220 |
| **Nausea** | 111 (15.3) | 17 (9.9) | 1 (16.7) | 129 (14.3) | 0.198 |
| **Vomiting** | 26 (3.6) | 2 (1.2) | 0 (0) | 28 (3.1) | 0.239 |
| **Mucus in the stool** | 23 (3.2) | 1 (0.6) | 1 (16.7) | 25 (2.8) | **0.021** |
| **Blood in the stool** | 6 (0.8) | 4 (2.3) | 0 (0) | 10 (1.1) | 0.227 |
| **Liquid stool** | 65 (8.9) | 20 (11.7) | 0 (0) | 85 (9.4) | 0.394 |
| **Intestinal sounds** | 124 (17.1) | 22 (12.9) | 2 (33.3) | 148 (16.4) | 0.218 |
| **Flatulence** | 196 (27.0) | 49 (28.7) | 3 (50.0) | 248 (27.4) | 0.418 |
| **Oral aphthae** | 82 (11.3) | 9 (5.3) | 0 (0) | 91 (10.1) | **0.045** |
| **Oral herpes** | 50 (6.9) | 11 (6.4) | 0 (0) | 61 (6.7) | 0.786 |
| **Dry eyes** | 156 (21.5) | 13 (7.6) | 2 (33.3) | 171 (18.9) | **0.000** |
| **Painful eyes** | 122 (16.8) | 19 (11.1) | 1 (16.7) | 142 (15.7) | 0.186 |
| **Conjunctivitis** | 31 (4.3) | 3 (1.8) | 0 (0) | 34 (3.8) | 0.266 |
| **Red eyes** | 44 (6.1) | 12 (7.0) | 0 (0) | 56 (6.2) | 0.733 |
| **Blurred vision** | 144 (19.8) | 25 (14.6) | 0 (0) | 169 (18.7) | 0.147 |
| **Diplopia** | 27 (3.7) | 4 (2.3) | 0 (0) | 31 (3.4) | 0.604 |
| **Photophobia** | 110 (15.1) | 13 (7.6) | 1 (16.7) | 124 (13.4) | **0.036** |
| **High Blood Pressure** | 68 (9.4) | 19 (11.1) | 0 (0) | 87 (9.6) | 0.567 |
| **Orthostatic hypotension** | 107 (14.7) | 12 (7.0) | 0 (0) | 119 (13.2) | **0.017** |
| **Tachycardia** | 220 (30.3) | 30 (17.5) | 1 (16.7) | 251 (27.8) | **0.003** |
| **Bradycardia** | 25 (3.4) | 5 (2.9) | 0 (0) | 30 (3.3) | 0.851 |
| **Arthritis (joint inflammation)** | 55 (7.6) | 1 (0.6) | 1 (16.7) | 57 (6.3) | **0.002** |
| **Neck pain** | 161 (22.1) | 26 (15.2) | 1 (16.7) | 188 (20.8) | 0.128 |
| **Right hypochondrium pain** | 108 (14.9) | 9 (5.3) | 2 (33.3) | 119 (13.2) | **0.001** |
| **Left hypochondrium pain** | 75 (10.3) | 14 (8.2) | 1 (16.7) | 90 (10.0) | 0.605 |
| **Pain from old injuries** | 86 (11.8) | 18 (10.5) | 0 (0) | 104 (11.5) | 0.602 |
| **Pins and needles pain** | 127 (17.5) | 16 (9.4) | 1 (16.7) | 144 (15.9) | **0.033** |
| **Rib pain** | 100 (13.8) | 20 (11.7) | 0 (0) | 120 (13.3) | 0,488 |
| **Ageusia** | 118 (16.2) | 12 (7.0) | 0 (0) | 130 (14.4) | **0.005** |
| **Anosmia** | 158 (21.7) | 24 (14.0) | 0 (0) | 182 (20.1) | **0.036** |
| **Cacosmia** | 129 (17.7) | 17 (9.9) | 0 (0) | 146 (16.2) | **0.025** |
| **Phantosmia** | 109 (15.0) | 15 (8.8) | 0 (0) | 124 (13.7) | 0.064 |
| **Loss of hearing** | 99 (13.6) | 9 (5.3) | 1 (16.7) | 109 (12.1) | **0.010** |
| **Excessive hearing** | 98 (13.5) | 12 (7.0) | 0 (0) | 110 (12.2) | **0.044** |
| **Hypoesthesia** | 58 (8.0) | 13 (7.6) | 1 (16.7) | 72 (8.0) | 0.722 |
| **Cramps** | 127 (17.5) | 26 (15.2) | 2 (33.3) | 155 (17.1) | 0.446 |
| **Fasciculations** | 95 (13.1) | 16 (9.4) | 0 (0) | 111 (12.3) | 0.270 |
| **Incoordination** | 64 (8.8) | 10 (5.8) | 0 (0) | 74 (8.2) | 0.342 |
| **Difficulty in fine motor skills** | 76 (10.5) | 13 (7.6) | 0 (0) | 89 (9.8) | 0.381 |
| **Disorientation** | 95 (13.1) | 9 (5.3) | 1 (16.7) | 105 (11.6) | **0.015** |
| **Anomia** | 281 (38.7) | 42 (24.6) | 1 (16.7) | 324 (35.8) | **0.002** |
| **Alexia** | 99 (13.6) | 8 (4.7) | 0 (0) | 107 (11.8) | **0.003** |
| **Trembling** | 54 (7.4) | 8 (4.7) | 1 (16.7) | 63 (7.0) | 0.288 |
| **Convulsions** | 3 (0.4) | 1 (0.6) | 0 (0) | 4 (0.4) | 0.942 |
| **Onset insomnia** | 220 (30.3) | 38 (22.2) | 2 (33.3) | 260 (28.8) | 0.109 |
| **Maintenance insomnia** | 289 (39.8) | 54 (31.6) | 4 (66.7) | 347 (38.4) | 0.051 |
| **Hair loss** | 232 (31.9) | 17 (9.9) | 2 (33.3) | 251 (27.8) | **0.000** |
| **Increased body odour** | 85 (11.7) | 14 (8.2) | 0 (0) | 99 (11.0) | 0.288 |
| **Dry skin** | 219 (30.1) | 21 (12.3) | 2 (33.3) | 242 (26.8) | **0.000** |
| **Itchy skin** | 155 (21.3) | 27 (15.8) | 1 (16.7) | 183 (20.2) | 0.263 |
| **Rash on the skin** | 98 (13.5) | 17 (9.9) | 0 (0) | 115 (12.7) | 0.295 |
| **Erythema pernio** | 15 (2.1) | 5 (2.9) | 0 (0) | 20 (2.2) | 0.737 |
| **Livedo reticularis** | 15 (2.1) | 2 (1.2) | 0 (0) | 17 (1.9) | 0.699 |
| **Menstrual alterations** | 121 (16.6) | 1 (0.6) | 0 (0) | 122 (13.5) | **0.000** |
| **Changes in the lenght of the cycle** | 86 (11.8) | 1 (0.6) | 0 (0) | 87 (9.6) | **0.000** |
| **Changes in the volume of the cycle** | 73 (10.0) | 1 (0.6) | 0 (0) | 74 (8.2) | **0.000** |
| **Dysmenorrhea** | 101 (13.9) | 1 (0.6) | 0 (0) | 102 (11.3) | **0.000** |
| **Vaginal discomfort** | 77 (10.6) | 0 (0) | 1 (16.7) | 78 (8.6) | **0.000** |
| **Genital discomfort** | 2 (0.3) | 9 (5.3) | 0 (0) | 11 (1.2) | **0.000** |
| **Dysuria** | 54 (7.4) | 5 (2.9) | 1 (16.7) | 60 (6.6) | 0.064 |
| **Frequent micturition** | 115 (15.8) | 25 (14.6) | 2 (33.3) | 142 (15.7) | 0.457 |
| **Low sexual desire** | 204 (28.1) | 36 (21.1) | 2 (33.3) | 242 (26.8) | 0.165 |
| **Sexual Disfunction** | 74 (10.2) | 7 (4.1) | 2 (33.3) | 83 (9.2) | **0.006** |
