# Supplementary material for: Characterisation, symptom pattern and symptom clusters from a retrospective cohort of Long COVID patients in primary care in Catalonia
Source: BMC Infect Dis. 2024 Jan 15;24:82. doi: 10.1186/s12879-023-08954-x (PMC10789045; doi:10.1186/s12879-023-08954-x)
Supplement: Supplementary file 10 — Additional file 10: Table S6. Symptoms by system by sex overtime. [file 12879_2023_8954_MOESM10_ESM.docx]

TABLE S6. Symptoms by system by sex overtime.

|  | Women | | | Men | | |
| --- | --- | --- | --- | --- | --- | --- |
|  | <21d | 22-60d | 3mesos | <21d | 22-60d | 3mesos |
| GENERAL | 675 (92.8) | 604 (83.1) | 579 (79.6) | 150 (87.2) | 143 (83.1) | 125 (72.7) |
| NEUROLOGICAL | 640 (88.0) | 583 (80.2) | 567 (78.0) | 135 (78.5) | 127 (73.8) | 128 (74.4) |
| RESPIRATORY | 601 (82.7) | 509 (70.0) | 433 (59.5) | 136 (79.0) | 125 (72.7) | 93 (54.0) |
| RHEUMATOLOGIC | 537 (73.9) | 483 (66.4) | 485 (66.7) | 105 (61.0) | 103 (59.9) | 92 (53.5) |
| UPPER RESPIRATORY WAYS | 519 (71.4) | 399 (54.9) | 371 (51.0) | 111 (64.5) | 99 (57.5) | 86 (50) |
| CARDIOLOGIC | 494 (67.9) | 462 (63.5) | 402 (55.3) | 94 (54.6) | 95 (55.2) | 79 (45.9) |
| DERMATOLOGIC | 349 (48.0) | 421 (57.9) | 379 (52.1) | 42 (24.4) | 58 (33.7) | 49 (28.5) |
| OLFACTORY | 440 (60.5) | 344 (47.3) | 254 (34.9) | 76 (44.2) | 54 (31.4) | 36 (20.9) |
| DIGESTIVE | 423 (58.2) | 354 (48.7) | 320 (44.0) | 88 (51.2) | 76 (44.2) | 70 (40.7) |
| NEUROCOGNITIVE | 399 (54.9) | 453 (62.3) | 510 (70.1) | 78 (45.3) | 95 (55.2) | 96 (55.8) |
| TASTE/SMELL | 366 (50.3) | 229 (31.5) | 106 (14.6) | 56 (32.5) | 30 (17.4) | 11 (6.4) |
| ENT OTHERS | 258 (35.5) | 285 (39.2) | 286 (39.3) | 53 (30.8) | 56 (32.5) | 51 (29.6) |
| OPHTALMOLOGIC | 309 (42.5) | 290 (39.9) | 287 (39.5) | 55 (32) | 60 (34.9) | 47 (27.3) |
| DYSAUTONOMIC | 230 (31.6) | 233 (32.0) | 219 (30.1) | 61 (35.4) | 54 (31.4) | 41 (23.8) |
| SEXUAL | 195 (26.8) | 197 (27.1) | 219 (30.1) | 48 (27.9) | 41 (23.8) | 38 (22.1) |
| GYNAECOLOGICAL | 138 (19) | 178 (24.5) | 191 (26.3) | 1 (0.6) | 1 (0.6) | 1 (0.58) |
| MENSTRUAL CYCLE | 115 (15.8) | 146 (20.1) | 160 (22.0) | 1 (0.6) | 1 (0.6) | 1 (0.58) |
| UROLOGIC | 54 (7.4) | 59 (8.1) | 56 (7.7) | 14 (8.1) | 10 (5.8) | 11 (6.4) |

ENT : Ear, Nose and Throat

Ordered by frequency of women’s symptoms at baseline.
