# Supplementary material for: Characterisation, symptom pattern and symptom clusters from a retrospective cohort of Long COVID patients in primary care in Catalonia
Source: BMC Infect Dis. 2024 Jan 15;24:82. doi: 10.1186/s12879-023-08954-x (PMC10789045; doi:10.1186/s12879-023-08954-x)
Supplement: Supplementary file 11 — Additional file 11: Table S7. Symptoms by system by age. [file 12879_2023_8954_MOESM11_ESM.docx]

Table S7. Symptoms by system by age.

|  | **18-34 yo (N=127)** | | | **35-49 yo (N=432)** | | | **50-64 yo (N=298)** | | | **≥64 yo (N=47)** | | |
| --- | --- | --- | --- | --- | --- | --- | --- | --- | --- | --- | --- | --- |
|  | **Baseline** | **22-60d** | **≥3months** | **Baseline** | **22-60d** | **≥3months** | **Baseline** | **22-60d** | **≥3months** | **Baseline** | **22-60d** | **≥3months** |
| **DERMATOLOGIC** | 52 (40.9) | 65 (51.2) | 53 (41.7) | 186 (43.0) | 234 (54.1) | 204 (47.2) | 134 (44.9) | 161 (54.0) | 157 (52.7) | 21 (44.7) | 22 (46.8) | 17 (36.1) |
| **OPHTALMOLOGIC** | 42 (33.1) | 38 (29.9) | 43 (33.8) | 169 (39.1) | 177 (40.9) | 156 (36.1) | 138 (46.3) | 124 (41.6) | 121 (40.6) | 17 (36.1) | 13 (27.6) | 16 (34.0) |
| **GYNAECOLOGICAL** | 28 (22.0) | 36 (28.3) | 37 (29.1) | 86 (19.9) | 113 (26.1) | 128 (29.6) | 25 (8.4) | 29 (9.7) | 27 (9.0) | 0 (0) | 1 (2.1) | 1 (2.1) |
| **MENSTRUAL CYCLE** | 26 (20.5) | 32 (25.2) | 35 (27.5) | 77 (17.8) | 103 (23.8) | 112 (25.9) | 13 (4.3) | 12 (4.0) | 14 (4.7) | 0 (0) | 0 (0) | 0 (0) |
| **UROLOGIC** | 10 (7.9) | 11 (8.6) | 6 (4.7) | 31 (7.1) | 37 (8.5) | 40 (9.2) | 25 (8.4) | 21 (7.0) | 21 (7.0) | 4 (8.5) | 2 (4.2) | 1 (2.1) |
| **SEXUAL** | 41 (32.3) | 38 (29.9) | 39 (30.7) | 115 (26.6) | 116 (26.8) | 126 (29.1) | 80 (26.8) | 78 (26.2) | 83 (27.8) | 8 (17.0) | 7 (14.9) | 11 (23.4) |
| **DIGESTIVE** | 74 (58.3) | 61 (48.0) | 59 (46.4) | 245 (56.7) | 205 (47.4) | 185 (42.8) | 173 (58.0) | 146 (48.9) | 130 (43.6) | 22 (46.8) | 20 (42.5) | 19 (40.4) |
| **UPPER RESPIRATORY AIRWAYS** | 85 (66.9) | 63 (49.6) | 55 (43.3) | 298 (68.9) | 230 (53.2) | 209 (48.4) | 221 (74.1) | 184 (61.7) | 168 (56.4) | 30 (63.8) | 24 (51.0) | 27 (57.4) |
| **OLFACTORY** | 87 (68.5) | 68 (53.5) | 50 (39.4) | 230 (53.2) | 183 (42.3) | 131 (30.3) | 177 (59.4) | 133 (44.6) | 100 (33.5) | 25 (53.2) | 16 (34.0) | 9 (19.1) |
| **EARS, NOSE AND THROAT (OTHERS)** | 44 (34.6) | 48 (37.8) | 42 (33.1) | 136 (31.5) | 164 (37.9) | 153 (35.4) | 116 (38.9) | 118 (39.6) | 123 (41.3) | 16 (34.0) | 12 (25.5) | 20 (42.5) |
| **RESPIRATORY** | 104 (81.9) | 89 (70.1) | 72 (56.7) | 356 (82.4 ) | 311 (72) | 268 (62.0) | 249 (83.5) | 213 (71.5) | 168 (56.4) | 34 (72.3) | 24 (51.0) | 20 (42.5) |
| **CARDIAC** | 84 (66.1) | 75 (59.0) | 67 (52.7) | 279 (64.6) | 279 (64.6) | 237 (54.8) | 200 (67.1) | 184 (61.7) | 160 (53.7) | 28 (59.6) | 23 (48.9) | 21 (44.7) |
| **RHEUMATOLOGIC** | 89 (70.1) | 71 (55.9) | 64 (50.4) | 318 (73.6) | 293 (67.8) | 290 (67.1) | 212 (71.1) | 204 (68.4) | 199 (66.7) | 27 (57.4) | 22 (46.8) | 28 (59.6) |
| **GENERAL (INCLUDES FATIGUE)** | 116 (91.3) | 102 (80.3) | 95 (74.8) | 402 (93.0) | 367 (84.9) | 346 (80.1) | 273 (91.6) | 252 (84.5) | 235 (78.8) | 40 (85.1) | 31 (65.9) | 32 (68.1) |
| **NEUROLOGICAL (INCLUDES HEADACHE AND INSOMNIA)** | 110 (86.6) | 93 (73.2) | 90 (70.8) | 375 (86.8) | 355 (82.2) | 336 (77.7) | 263 (88.2) | 240 (80.5) | 243 (81.5) | 32 (68.1) | 27 (57.4) | 30 (63.8) |
| **NEUROCOGNITIVE** | 54 (42.5) | 67 (52.7) | 74 (58.2) | 222 (51.4) | 269 (62.2) | 298 (68.9) | 183 (61.4) | 193 (64.7) | 208 (69.8) | 22 (46.8) | 22 (46.8) | 29 (61.7) |
| **DISAUTONOMIC** | 41 (32.3) | 43 (33.8) | 37 (29.1) | 134 (31.0) | 136 (31.5) | 115 (26.6) | 106 (35.6) | 100 (33.5) | 102 (34.2) | 13 (27.6) | 10 (21.3) | 8 (17.0) |
| **TASTE AND SMELL** | 75 (59.0) | 50 (39.4) | 27 (21.2) | 191 (44.2) | 117 (27.1) | 50 (11.6) | 138 (46.3) | 80 (26.8) | 36 (12.1) | 20 (42.5) | 13 (27.6) | 4 (8.5) |

Joves:

- baseline generals, nrl, respi, reuma i olfactoris.
- Actuals: generals, neuro, respi i neurocogn

Mitjana edat

- Baseline: generals, neuro, respi, reuma, upper respiratoryi olfactoris
- Actuals: general, neurològic i neurocognitius, seguits de reuma.

50-64a

- Baseline: generals, neuro, respiratori, upper respiratory i reuma
- Actuals: neuro, general, neurocogn, reumatologics

+64a

- Baseline: general, respiratori, neurològic, upper respiratory ways, cardíacs
- Actuals: generals, neuro, neurocognitius, reuma i upper respiratory ways
