# Supplementary material for: Characterisation, symptom pattern and symptom clusters from a retrospective cohort of Long COVID patients in primary care in Catalonia
Source: BMC Infect Dis. 2024 Jan 15;24:82. doi: 10.1186/s12879-023-08954-x (PMC10789045; doi:10.1186/s12879-023-08954-x)
Supplement: Supplementary file 12 — Additional file 12: Table S8. Symptoms by system by wave over time. [file 12879_2023_8954_MOESM12_ESM.docx]

**TABLE S8**. Symptoms by system by wave over time.

| **Symptoms by system** | **Baseline** | | | |  | **22-60 days** | | | |  | **≥ 3 months** | | | |  |
| --- | --- | --- | --- | --- | --- | --- | --- | --- | --- | --- | --- | --- | --- | --- | --- |
|  | **First wave** | **Second wave** | **Third wave** | **Fourth wave** | **Total (N=905)** | **First wave** | **Second wave** | **Third wave** | **Fourth wave** | **Total (N=905)** | **First wave** | **Second wave** | **Third wave** | **Fourth wave** | **Total**  **N=905** |
| **DermatologiC** | 197 (44.6) | 110 (43.5) | 17 (41.5) | 1 (100) | 325 (35.9) | 253 (57.2) | 120 (47.4) | 15 (36.6) | 1 (100) | 389 (43) | 206 (46.6) | 122 (48.2) | 18 (43.9) | 1 (100) | 347 (38.3) |
| **Ophtalmologic** | 181 (40.9) | 93 (36.7) | 17 (41.5) | 1 (100) | 292 (32.2) | 177 (40.0) | 86 (34) | 14 (34.1) | 1 (100) | 278 (30.7) | 170 (38.4) | 84 (33.2) | 13 (31.7) | 1 (100) | 268 (29.6) |
| **Gyneacological** | 70 (15.8) | 34 (13.4) | 8 (19.5) | 1 (100) | 113 (12.5) | 91 (20.6) | 39 (15.4) | 9 (21.9) | 1 (100) | 140 (15.4) | 98 (22.2) | 42 (16.6) | 11 (26.8) | 1 (100) | 152 (16.8) |
| **Menstrual cycle** | 59 (13.3) | 29 (11.4) | 7 (17.1) | 1 (100) | 96 (10.6) | 73 (16.5) | 32 (12.6) | 9 (21.9) | 1 (100) | 115 (12.7) | 81 (18.3) | 36 (14.2) | 11 (26.8) | 1 (100) | 129 (14.2) |
| **Urologic** | 28 (6.3) | 21 (8.3) | 4 (9.7) | 1 (100) | 54 (5.9) | 33 (7.4) | 18 (7.1) | 3 (7.3) | 0 (0) | 54 (5.9) | 28 (6.3) | 16 (6.3) | 2 (4.8) | 0 (0) | 46 (5.1) |
| **Sexual** | 111 (25.1) | 67 (26.5) | 15 (36.6) | 1 (100) | 194 (21.4) | 107 (24.2) | 59 (23.3) | 10 (24.4) | 1 (100) | 177 (19.5) | 120 (27.1) | 62 (24.5) | 12 (29.2) | 1 (100) | 195 (21.5) |
| **Digestive** | 266 (60.2) | 135 (53.3) | 22 (53.6) | 1 (100) | 424 (46.8) | 223 (50.4) | 99 (39.1) | 17 (41.5) | 1 (100 ) | 340 (37.6) | 195 (44.1) | 88 (34.8) | 21 (51.2) | 1 (100) | 305 (33.7) |
| **Upper respiratory ways** | 300 (67.9) | 176 (69.5) | 29 (70.7) | 1 (100) | 506 (55.9) | 240 (54.3) | 128 (50.6) | 22 (53.5) | 1 (100) | 391 (43.2) | 209 (47.3) | 131 (51.8) | 21 (51.2) | 1 (100) | 362 (40.0) |
| **Olfactory** | 241 (54.5) | 149 (58.9) | 26 (63.4) | 1 (100) | 417 (46.1) | 197 (44.6) | 197 (77.8) | 18 (43.9) | 1 (100) | 323 (35.7) | 144 (32.6) | 74 (29.2) | 12 (29.2) | 1 (100) | 231 (25.5) |
| **Ear, nose, Throat others** | 147 (33.2) | 89 (35.2) | 14 (34.1) | 1 (100) | 251 (27.7) | 164 (37.1) | 92 (36.3) | 15 (36.6) | 1 (100) | 272 (30.0) | 162 (36.6) | 88 (34.8) | 17 (41.5) | 1 (100) | 268 (29.6) |
| **Respiratory** | 364 (82.3) | 204 (80.6) | 31 (75.6) | 1 (100) | 600 (66.3) | 315 (71.2) | 176 (69.5) | 21 (51.2) | 1 (100) | 513 (56.7) | 252 (57.0) | 146 (57.7) | 22 (53.6) | 1 (100) | 421 (46.5) |
| **Cardiac** | 294 (66.5) | 162 (64.0) | 26 (63.4) | 1 (100) | 483 (53.4) | 286 (64.7) | 139 (54.9) | 21 (51.2) | 1 (100) | 447 (49.4) | 246 (55.6) | 123 (48.6) | 21 (51.2) | 1 (100) | 391 (43.2) |
| **Rheumatologic** | 312 (70.6) | 179 (70.7) | 30 (73.1) | 1 (100) | 522 (57.7) | 293 (66.3) | 155 (61.2) | 23 (56.1) | 1 (100) | 472 (52.1) | 283 (64.0) | 162 (64.0) | 24 (58.5) | 1 (100) | 470 (51.9) |
| **Generals** | 403 (91.1) | 230 (90.9) | 38 (92.7) | 1 (100) | 672 (74.2) | 366 (82.8) | 202 (79.8) | 32 (78.0) | 1 (100) | 601 (66.4) | 346 (78.3) | 189 (74.7) | 33 (80.5) | 1 (100) | 569 (62.9) |
| **Neurological (headache and insomina included)** | 380 (85.9) | 215 (85) | 35 (85.3) | 1 (100) | 631 (69.7) | 353 (79.8) | 193 (76.3) | 31 (75.6) | 1 (100) | 578 ( 63.8) | 334 (75.5) | 192 (75.9) | 33 (80.5) | 1 (100) | 560 (61.9) |
| **Neurocognitive** | 226 (51.1) | 140 (55.3) | 28 (68.3) | 1 (100) | 395 (43.6) | 274 (61.9) | 148 (58.5) | 22 (53.5) | 1 (100) | 445 (49.2) | 300 (67.9) | 162 (64.0) | 24 (58.5) | 1 (100) | 487 (53.8) |
| **Disautonomic** | 146 (33.0) | 82 (32.4) | 13 (31.7) | 1 (100) | 242 (26.7) | 144 (32.6) | 71 (28.1) | 12 (29.2) | 1 (100) | 228 (25.2) | 125 (28.3) | 70 (27.6) | 11 (26.8) | 1(100) | 207 (22.9) |
| **Taste and smell** | 192 (43.4) | 132 (52.2) | 20 (48.8) | 1 (100) | 345 (38.1) | 117 (26.5) | 80 (31.6) | 11 (26.8) | 0 (0) | 208 (22.9) | 51 (11.5) | 40 (15.8) | 5 (12.2) | 0 (0) | 96 (10.6) |

First wave N= 442

Second wave N=253

Third wave N= 41

Fourth wave N=1

N total de les onades 737 (168 missings)

N total de respondents 905
