# Supplementary material for: Characterisation, symptom pattern and symptom clusters from a retrospective cohort of Long COVID patients in primary care in Catalonia
Source: BMC Infect Dis. 2024 Jan 15;24:82. doi: 10.1186/s12879-023-08954-x (PMC10789045; doi:10.1186/s12879-023-08954-x)
Supplement: Supplementary file 13 — Additional file 13: Table S9. Symptoms by system by PCR or RAT result. [file 12879_2023_8954_MOESM13_ESM.docx]

**TABLE S9.** Symptoms by system by PCR or RAT result.

| SYMPTOMS SORTED BY SYSTEM | TEST result | | | | | | | | |
| --- | --- | --- | --- | --- | --- | --- | --- | --- | --- |
|  | POSITIVE AT ANY TIME  N (%) N=**464 (51.5)** | | | NEVER POSITIVE N (%)  N=437 (48.5) | | | Total N (%) N=901 (4 missings) | | |
|  | Baseline | 22-60 days | ≥3months | Baseline | 22-60 days | ≥3months | Baseline | 22-60 days | ≥3months |
| DermatolOGIC | 212 (45.7) | 252 (54.3) | 220 (47.4) | 179 (40.9) | 228 (52.2) | 211 (48.3) | 391 (43.2) | 480 (53.0) | 431 (47.6) |
| OPHtalmolOGIC | 185 (39.9) | 173 (37.3) | 166 (35.8) | 181 (41.4) | 179 (40.9) | 170 (38.9) | 366 (40.4) | 352 (38.9) | 336 (37.1) |
| GYNAECOLOGICal | 67 (14.4) | 85 (18.3) | 86 (18.5) | 71 (16.2) | 93 (21.3) | 106 (24.2) | 138 (15.2) | 178 (19.7) | 192 (21.2) |
| menstrual cycle | 56 (12.1) | 68 (14.3) | 72 (15.5) | 59 (13.5) | 78 (17.8) | 88 (20.1) | 115 (12.7) | 146 (16.1) | 160 (17.7) |
| UrolOGIC | 37 (7.9) | 35 (7.5) | 29 (6.2) | 33 (7.5) | 36 (8.2) | 39 (8.9) | 70 (7.7) | 71 (7.8) | 68 (7.5) |
| SEXUAL | 136 (29.3) | 119 (25.6) | 127 (27.4) | 107 (24.5) | 119 (27.2) | 131 (29.9) | 243 (26.9) | 238 (26.3) | 258 (28.5) |
| DIGESTIVE | 282 (60.8) | 215 (46.3) | 191 (41.2) | 231 (52.9) | 215 (49.2) | 202 (46.2) | 513 (56.7) | 430 (47.5) | 393 (43.4) |
| UPPER RESPIRATORY AIRWAYS | 333 (71.8) | 244 (52.6) | 228 (49.1) | 299 (68.4) | 256 (58.6) | 230 (52.6) | 632 (69.8) | 500 (55.2) | 458 (50.6) |
| OLFACTORY | 295 (63.6) | 216 (46.5) | 147 (31.7) | 222 (50.8) | 182 (41.6) | 142 (32.5) | 517 (57.1) | 398 (44.0) | 289 (31.9) |
| ears, nose and throat (OTHERS) | 162 (34.9) | 169 (36.4) | 167 (36) | 149 (34.1) | 172 (39.3) | 171 (39.1) | 311 (34.4) | 341 (37.7) | 338 (37.3) |
| RESPIRATORY | 391 (84.3) | 334 (71.9) | 271 (58.4) | 351 (80.3) | 302 (69.1) | 257 (58.8) | 742 (82) | 636 (70.3) | 528 (58.3) |
| CardIAC | 314 (67.7) | 284 (61.2) | 237 (51.1) | 276 (63.1) | 275 (62.9) | 247 (56.5) | 590 (65.2) | 559 (61.8) | 484 (53.5) |
| RHeumatOLOGic | 348 (75) | 308 (66.4) | 300 (64.6) | 297 (67.9) | 280 (64.1) | 280 (64.1) | 645 (71.3) | 588 (65.0) | 580 (64.1) |
| General (INCLUDES FATIGUE) | 435 (93.7) | 398 (85.8) | 370 (79.7) | 393 (89.9) | 351 (80.3) | 337 (77.1) | 828 (91.5) | 749 (82.8) | 707 (78.1) |
| NEUROLOGICal (INCLUDES HEADACHE AND INSOMNIA) | 416 (89.6) | 376 (81.0) | 370 (79.7) | 362 (82.8) | 337 (77.1) | 328 (75) | 778 (86.0) | 713 (78.8) | 698 (77.1) |
| NeurocognitiVE | 266 (57.3) | 293 (63.1) | 320 (68.9) | 214 (48.9) | 256 (58.6) | 288 (65.9) | 480 (53.0) | 549 (60.7) | 608 (67.2) |
| Disautonomic | 156 (33.6) | 144 (31.0) | 123 (26.5) | 138 (31.6) | 145 (33.2) | 139 (31.8) | 294 (32.5) | 289 (31.9) | 262 (29.0) |
| TASTE AND SMELL | 250 (53.9) | 148 (31.9) | 66 (14.2) | 172 (39.3) | 112 (25.6) | 51 (11.7) | 422 (46.6) | 260 (28.7) | 117 (12.9) |
